# Humans monitor learning progress in curiosity-driven exploration

**DOI:** 10.1038/s41467-021-26196-w

**Published:** 2021-10-13

**Authors:** Alexandr Ten, Pramod Kaushik, Pierre-Yves Oudeyer, Jacqueline Gottlieb

**Affiliations:** 1 INRIA Bordeaux Sud-Ouest, 200 Avenue de la Vieille Tour, 33405 Talence, France; 2 Department of Neuroscience & The Kavli Institute for Brain Science, Columbia University, 1051 Riverside Drive, Kolb Research Annex, Rm. 569, New York, NY 10032 USA

**Keywords:** Psychology, Human behaviour

## Abstract

Curiosity-driven learning is foundational to human cognition. By enabling humans to autonomously decide when and what to learn, curiosity has been argued to be crucial for self-organizing temporally extended learning curricula. However, the mechanisms driving people to set intrinsic goals, when they are free to explore multiple learning activities, are still poorly understood. Computational theories propose different heuristics, including competence measures (e.g., percent correct) and learning progress, that could be used as intrinsic utility functions to efficiently organize exploration. Such intrinsic utilities constitute computationally cheap but smart heuristics to prevent people from laboring in vain on unlearnable activities, while still motivating them to self-challenge on difficult learnable activities. Here, we provide empirical evidence for these ideas by means of a free-choice experimental paradigm and computational modeling. We show that while humans rely on competence information to avoid easy tasks, models that include a learning-progress component provide the best fit to task selection data. These results bridge the research in artificial and biological curiosity, reveal strategies that are used by humans but have not been considered in computational research, and introduce tools for probing how humans become intrinsically motivated to learn and acquire interests and skills on extended time scales.

## Abstract (web summary)

Mechanisms underlying time-extended, curiosity-driven learning in humans are still poorly understood. Here, the authors present empirical evidence that humans rely on learning progress in deciding how to allocate time in free exploration of multiple learning activities.

## Introduction

Curiosity, our desire to know, is a fundamental drive in human behavior and a topic of renewed interest in neuroscience and cognitive psychology^1,2^. The vast majority of recent research on curiosity has operationalized it as intrinsically motivated information demand, using tasks in which participants can request information about future events but do not have the opportunity to exploit (act on) the information. The studies have shown that humans and other animals seek to obtain information as a good in itself and this preference is encoded in neural systems of reward and motivation, suggesting that information is rewarding independently of material gains^3–6^.

While these findings tap into the intrinsic motivation behind curiosity, they are yet to capture the full scope of curiosity-driven investigations^7^. Specifically, in natural settings, humans investigate questions on much longer time scales relative to those tested in the laboratory. In contrast with tasks of information demand in which participants request information about brief unrelated events – e.g., a forthcoming reward or a trivia question – in natural behavior, learners maintain sustained focus on specific activities such as reading an article, conducting an online search, or taking a course. Operating from early infant development^8^, this ability for sustained investigations may underlie the most important ecological role of curiosity, as it allows people to develop individual interests and skills and, ultimately, discover explanatory models and latent structures of the world^9–11^.

Very little is known about how people self-organize investigations to achieve learning on longer time scales. Natural environments afford a practically infinite number of activities that a curious learner can in principle investigate. However, given the limited time and resources available for investigation, the learner must carefully select which activity to engage with to enable discovery. Formal treatment of this “strategic student” problem prescribe how learners should allocate study time to maximize learning across a set of the activities^12,13^ but show that the optimal allocation is very sensitive to the shape of the expected learning trajectory, which is not available to learners in practice^12^.

A common proposal for how people resolve this conundrum is that they prioritize study items based on their perceived difficulty, i.e., their perceived level of knowledge or competence on a task, but the precise form of this prioritization is under debate. Several studies have shown that people prioritize tasks with high difficulty or high uncertainty^14,15^. In contrast, an expanding literature proposes that people prefer intermediate difficulty^16^ in a range of conditions including curiosity about trivia questions^5,17^, choices among sensorimotor activities^18^, infant attention^19^ and esthetic appreciation^20,21^.

Strategies that prioritize high versus intermediate difficulty activities may have different computational bases and ecological roles. A preference for high difficulty tasks may emerge from computational architectures that assign intrinsic utility to prediction errors or uncertainty, thus motivating agents to venture beyond familiar activities^15,22–24^. In contrast, a strategy prioritizing activities with intermediate difficulty may emerge from control architectures based on learning progress (LP)^25–30^ that monitor the temporal derivative of performance - e.g., percent correct (PC) - and generate intrinsic rewards for activities in which the agent’s performance changes with practice.

LP-based algorithms are particularly important in naturalistic environments because they allow agents to avoid not only highly familiar tasks but also unlearnable tasks – i.e., activities that are intrinsically random or cannot be mastered with the learners’ current knowledge or skills^30–32^. Unlike PC-based algorithms that steer agents toward tasks of maximum difficulty, LP-based algorithms help to avoid random or too-difficult activities. Moreover, these algorithms provide realistic solutions for optimizing study time allocation - by maximizing the progress that an agent experiences in practice without precise knowledge of one’s future learning curve^12,13^ - and have been applied to automate curriculum learning in difficult machine learning problems^27,33,34^ and personalize sequences of learning activities in educational technologies^35–37^.

Despite the potential importance of LP-based control strategies, there is no empirical evidence of whether, and how, people use such strategies. In the studies conducted so far, people were asked to estimate the difficulty of study materials based on their familiarity with the topic (e.g., biographical text or foreign vocabulary)^38^. However, no study has tested whether participants can dynamically monitor their performance on an arbitrary activity and use dynamic estimates of PC or its temporal derivative (LP) as predicted by computational algorithms.

Here we examined this question using computational modeling and a behavioral task in which people self-organized their study curricula based on trial-by-trial feedback about their performance on a set of novel activities. We provide direct evidence that humans show bona fide sensitivity to LP – the change in performance on novel activities – which coexists with a sensitivity to PC and steers people away from unlearnable tasks consistent with computational theories.

## Results

**Fig. 1:**
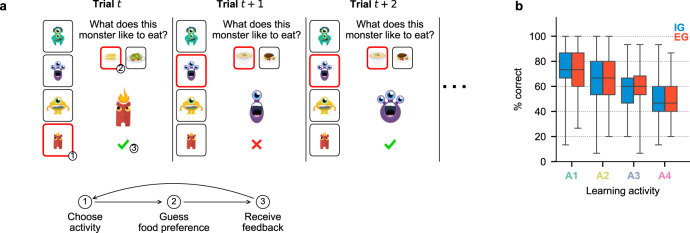
Task design and difficulty manipulation. **a** Trial structure during free play. The panels show 3 example free-choice trials consisting of 3 steps each. Each trial began with a choice among 4 "monster families" depicted as visual icons (1). This was followed by the presentation of a randomly drawn individual from that family and a prompt to guess which of two possible foods the individual liked to eat (2). After guessing (2), the participant received immediate feedback (3) and the next trial began. Participants were free to repeat the previously sampled activity (e.g., trial *t* + 2 in this figure) or switch to any other monster family (e.g., trial *t* + 1) as they wished. **b** Performance during the forced-choice familiarization stage. Each box plot shows the %correct (PC) during the 15 familiarization trials in which participants had to play each activity for the IG (blue; *N* = 186) and EG (red; *N* = 196) groups. Horizontal bars inside boxes show the median values across all participants in a group; box boundaries show the 1st and the 3rd quartiles; whiskers show sample minima and maxima. Image credits (**a**): monster character designs by macrovector/Freepik; food-item designs by brgfx/Freepik. Source data for **b** are provided as a Source Data file.

**Fig. 2:**
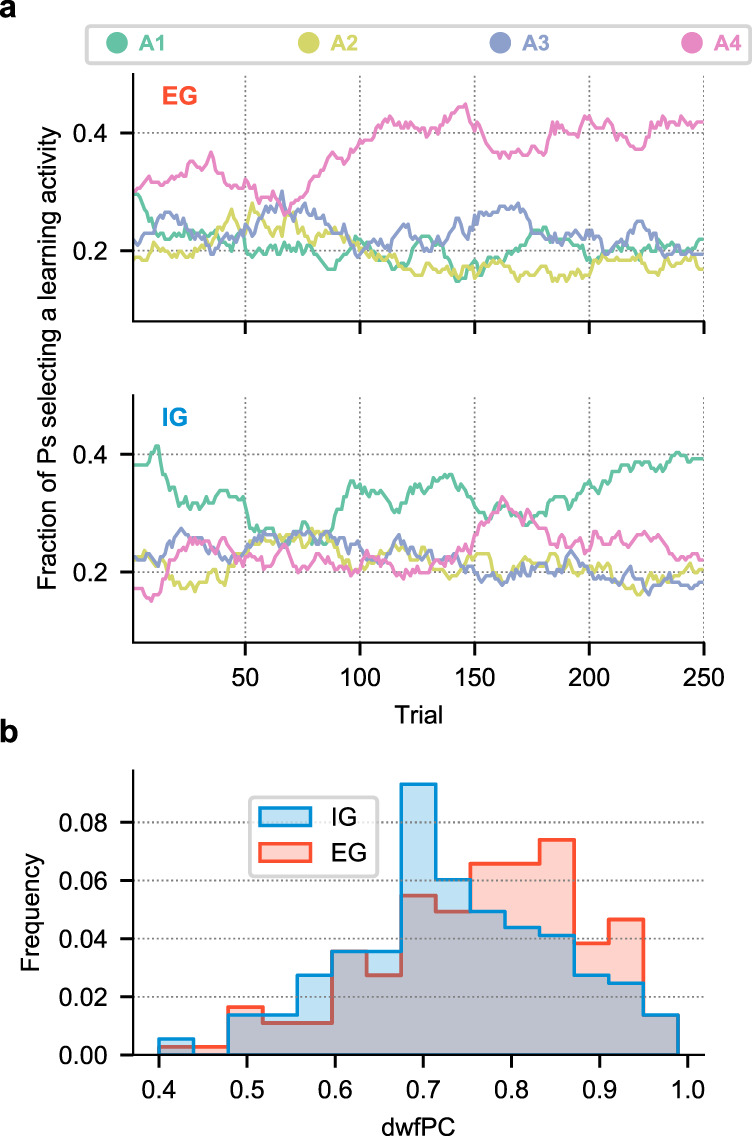
Free play behavior. **a** The fraction of participants selecting each learning activity in the EG (*N* = 196) and IG (*N* = 186) groups (respectively, top and bottom panels) as a function of trial number during the free play stage (no smoothing) demonstrate that group differences in choice patterns persisted throughout the task. **b** Histograms of difficulty-weighted final performance (dwfPC) for each instruction group. The EG group (*N* = 196) achieved better dwfPC scores than the IG group (*N* = 186), but the distributions were broad and overlapping, highlighting important individual variability. The difference between groups was significant with both dwfPC and unweighted average PC scores. Source data are provided as a Source Data file.

**Fig. 3:**
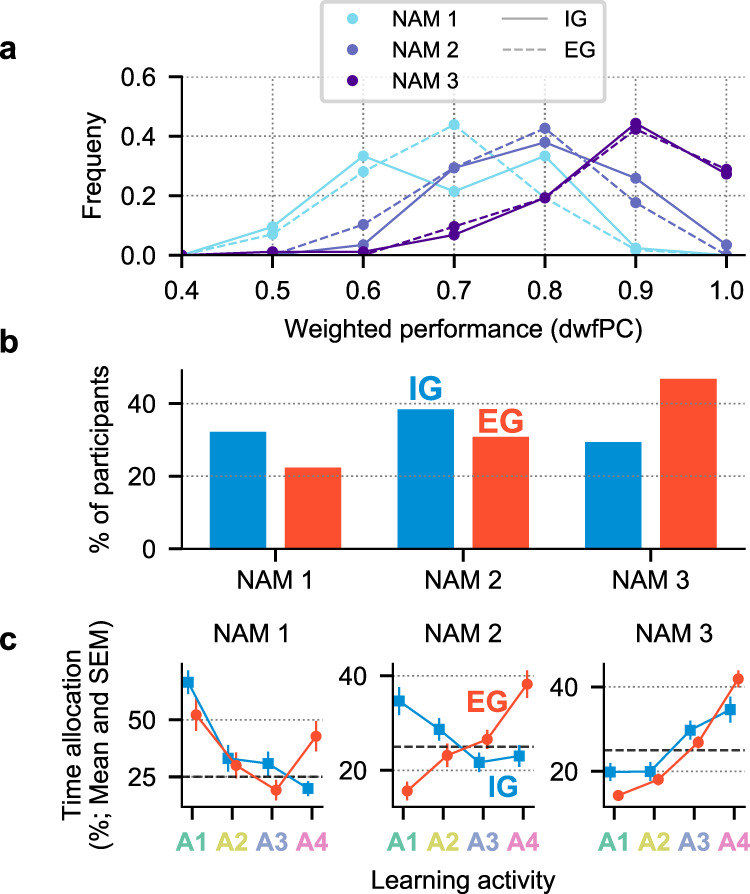
Individual variability within groups. **a** Final performance was the same across instruction groups when accounting for the number of activities mastered (NAM). As expected, the NAM designation captured well the learning achievement of our participants. In light of (**b**), this demonstrates that many participants achieved a high performance across learning activities, even without an explicit instruction to learn. **b** Distributions of participants mastering 1, 2, or 3 activities in each instruction group. Whereas half of the participants in the EG group achieved high performance across learnable tasks, a sizable portion of the IG participants (almost 1/3) were motivated enough to self-challenge and learn without being asked to do so. Only 8 participants in the EG and 9 participants in the IG group failed to master even one activity. Thus, 99 participants mastered only 1 activity (*N*_EG_ = 42; *N*_IG_ = 57), 126 mastered two (*N*_EG_ = 58; *N*_IG_ = 68), and 140 mastered all three (*N*_EG_ = 88; *N*_IG_ = 52) (**c**), Time allocation patterns differed by instruction and level of achievement. The three panels show the average time allocation patterns in IG (*N* = 177) and EG (*N* = 188) groups observed over the free-play trials separately for each level of NAM (from left to right, NAM1, NAM2, and NAM3). Circle (EG) and square (IG) symbols represent the average percentage of time spent on an activity in the respective NAM-instruction group; error bars indicate the standard error; the horizontal dashed lines show random time allocation (25%). Time allocation was consistent across the levels of NAM towards harder activities in the EG group. In contrast, only the best learners in the IG group displayed a similar preference, whereas NAM1&2 participants tended towards easier activities. Source data are provided as a Source Data file.

We analyzed data from 382 participants who performed an online task in which they could freely engage with a set of learning activities (Fig. 1a). Each trial started with a free-choice panel prompting the participant to choose one of 4 activities depicted as families of "monsters” (Fig. 1a, (1)). After making a choice, the participant received a randomly drawn member from the chosen family, made a binary guess about which food that member liked to eat (Fig. 1a, (2)), and received immediate feedback regarding their guess (Fig. 1a, (3)). To understand how participants self-organized their learning curriculum, we required them to complete 250 trials but did not impose any other constraint on their choice of activity.

Our key questions were (1) how people self-organize their exploration over a set of activities of variable difficulty, and (2) whether they spontaneously adopt learning maximization objectives when they do not receive explicit instructions. To examine these questions, we manipulated the difficulty of the available activities as a within-participant variable, and the instructions that participants received as an across-participant variable. Difficulty was controlled by the complexity of the categorization rule governing the food preferences. In the easiest activity (A1), individual monster-family members differed in only one feature and that feature governed their food preference (e.g., a red monster with big flame liked fries and a red monster with small flame liked salad; 1-dimensional categorization). In the next easiest level (A2), family members varied along two features but only one feature determined preference (1-dimensional with an irrelevant feature). In the most difficult learnable activity (A3) food preferences were determined by a conjunction of 2 variable features (2-dimensional categorization). Finally, the 4th activity (A4) was random and unlearnable: individual monsters had two variable features, but their food preferences were assigned randomly each time a new monster was sampled, and were thus unpredictable with either a rule-based or rote memorization strategy.

Learning objectives were manipulated across two randomly selected participant groups. Participants assigned to the “external goal” group (EG; *N* = 196) were asked to maximize learning across all the activities and were told that they will be tested at the end of the session. In contrast, participants in the “internal goal” group (IG, *N* = 186) were told to choose any activity they wished with no constraint except for completing 250 trials. Except for this difference in instructions (and the fact that the EG group received the announced test), the two groups received identical treatments. Each group started with 15 forced-choice familiarization trials on each activity, followed by a 250-trial free-play stage, and gave several subjective ratings of the activities before and after the free play stage (see Supplementary Fig. 2).

Performance on the forced-choice familiarization stage verified that these manipulations worked as intended. The EG and IG groups had equivalent performance during this stage (Fig. 1b; mixed-design ANOVA on percent correct (PC) with group and difficulty as factors; EG vs IG, *F*(1, 380) = 1.829, *p* = 0.177; group × difficulty interaction, *F*(3, 1140) = 0.820, *p* = 0.483). For both EG and IG participants, performance on each activity was significantly different from all others, suggesting that both groups could use performance feedback as an index of activity difficulty (Fig. 1b; mixed-design ANOVA, main effect of activity, *F*(3, 1140) = 158.400, *p* < 0.001; post-hoc pairwise Tukey’s HSD tests between all activity levels within each group were significant with all p-values smaller than *p* = 0.01). Additional evidence from the ratings obtained at the end of the task showed that the EG and IG groups provided similar retrospective ratings of time spent, progress made and interest in learning activities (Supplementary Fig. 2), suggesting that they had equivalent engagement and self-monitoring while performing the task.

### Individuals show spontaneous self-challenge independently of instructions

Despite their equivalent learning ability, EG and IG participants showed different choice patterns and substantial individual variability in the extent to which they challenged themselves and mastered the available tasks.

Analysis of group-level activity choices showed that, while the EG group focused strongly on the most difficult activity (the unlearnable activity that had the lowest PC), the IG group showed a more uniform preference with only a slight bias toward the easiest activity (Fig. 2, a). Across the entire session, the EG group had significant below-chance time allocation to the two easiest activities and above-chance allocation to the random (lowest-PC) activity (relative to 25%; linear model with sum contrasts: A1: 20.61%, *t*(1520) = −3.002, *p* = 0.003; A2: 19.29%; *t*(1520) = −3.910, *p* = 0.048; A4: 36.92%; *t*(1520) = 8.156, *p* < 0.001). In contrast, the IG group had a significant above-chance allocation for the easiest (A1) activity (A1: 33.00%, *t*(1520) = 5.330, *p* < 0.001) while spending less time on other activities (A2: 21.42%; *t*(1520) = −2.387, *p* = 0.017; A3: 22.16%; *p* > 0.05; A4: 23.43%; *p* > 0.05; Fig. 2, a). According to a significant interaction between instruction-group × activity-type interaction, revealed by a 2-way mixed design ANOVA of time allocation, these differences were reliable (*F*(3, 1140) = 14.578, *p* < 0.001).

Consistent with their higher self-challenge, average learning achieved by the end of the free-play stage was greater in the EG relative to the IG group (Fig. 2b). A measure of difficulty-weighted final PC (dwfPC: the average PC in the last 15 trials spent on each activity scaled by its difficulty rank (Methods, Difficulty-weighted final performance) was significantly higher for the EG group (M = 0.756, SD = 0.127) relative to the IG group (Fig. 2b; M = 0.721, SD = 0.126; *t*(379.4) = 2.679, *p* = 0.008, Welch two-sample *t*-test), and the same result held if we used unweighted average PC (EG: M = 0.787, SD = 0.118; IG M = 0.756, SD = 0.120; *t*(378.1) = 2.539, *p* = 0.011, Welch two-sample *t*-test).

Notwithstanding these group-level differences, participants showed substantial individual variability and, importantly, a subset of those in the IG group adopted levels of self-challenge similar to the EG group. To investigate this variability we categorized each participant based on the number of activities they mastered to a learning criterion - i.e., whether they mastered 1, 2 or all 3 learnable activities (NAM1, NAM2 or NAM3; see Methods, NAM designation). The dwfPC score within each NAM group was not affected by instructions, showing that the NAM designation effectively captured the variability in learning achievement (Fig. 3a; pairwise contrasts IG vs. EG conditioned on NAM were nonsignificant, *p* > 0.05, at all levels of NAM).

Importantly, despite not being instructed to study for a test, 64.52% of IG participants mastered more than one activity (NAM2 and NAM3) and 29.59% mastered all 3 activities (Fig. 3b). These percentages were comparable to learning achievements in the EG group, where 74.49% mastered at least 2 activities, and 36.56% mastered all three. The relative proportions of participants at each achievement level were comparable between the two groups across a range of mastery criteria (see Supplementary Fig. 3, for a detailed analysis). Thus, while changing the criterion modified the number of participants who achieved mastery, it left intact the relative fractions NAM subgroups in the IG and EG groups. This shows that our conclusions are independent from a specific definition of mastery.

While NAM1 and NAM2 participants in the IG group showed choices consistent with the group average – favoring the easiest activity – NAM3 participants showed a distinct preference for A3 and A4 activities that more closely resembled the EG group (Fig. 3c). Two-way mixed ANOVAs of time allocation showed in the IG group, a marginally significant main effect of activity (*F*(3, 525) = 8.847, *p* < 0.001) and a highly significant interaction between activity and NAM (*F*(3, 525) = 14.791, *p* < 0.001). In the EG group there was also a significant main effect of activity (*F*(3, 525) = 19.407, *p* < 0.001) and a significant interaction with NAM (*F*(3, 525) = 7.197, *p* < 0.001). As Fig. 3c shows, while participants in NAM1 and NAM2 groups differed in activity selection across the instruction conditions, those who mastered all 3 learnable activities allocated their time similarly. Importantly, a sizeable fraction of the IG group behaved in the same way as people who were instructed to learn and prepare for a test.

To further examine the relationship between learning achievement and activity choices, we created an index of self-challenge (SC) measuring the extent to which each participant tended to challenge themselves. This index was defined as the recent PC of the activity selected on each trial, normalized to the entire range of PC levels the participant experienced so far (Methods, Self-challenge index). Thus, SC values close to 0 denote participants who tended to choose the easiest of the activities they experienced; SC close to 1 denote participants who tended to choose the most difficult activities; and SC near 0.5 denote participants who preferred activities of intermediate difficulty. Supplementary analysis verified that the SC index is a more efficient measure of the tendency to choose challenging activities compared to simple contrasts between pairs of activities (Supplementary Fig. 4).

**Fig. 4:**
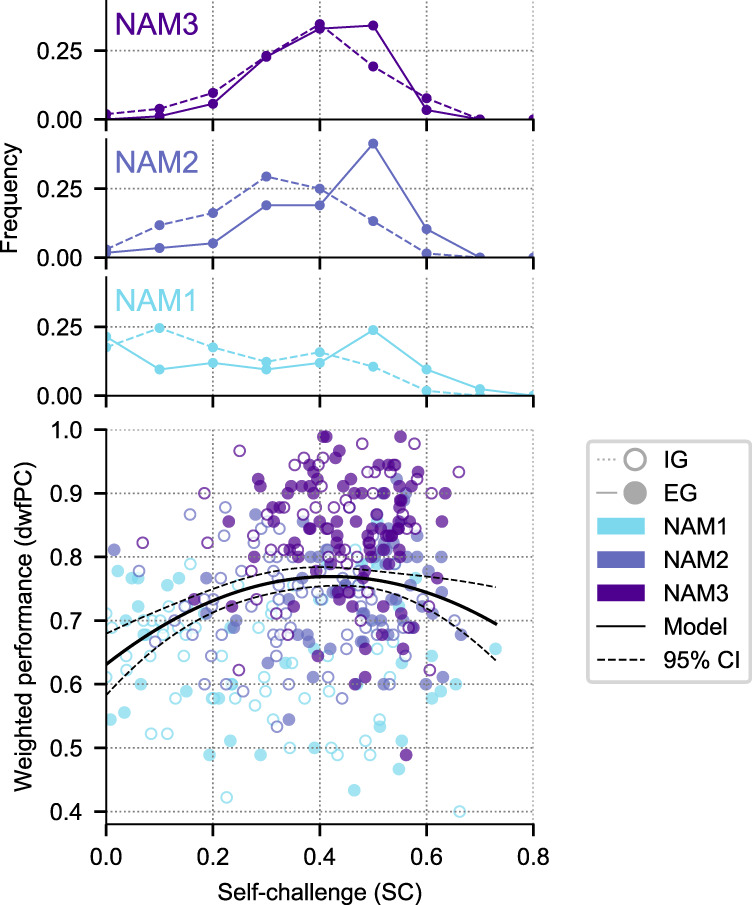
Relationship between activity choices and final performance. The scatter plot shows the difficulty-weighted final score (dwfPC; *y*-axis) as a function of the self-challenge index (SC; *x*-axis). Each point is one participant. Colors indicate the number of activities mastered: NAM1, *N* = 99 (*N*_EG_ = 42; *N*_IG_ = 57); NAM2, *N* = 126 (*N*_EG_ = 58; *N*_IG_ = 68); and NAM3, *N* = 140 (*N*_EG_ = 88; *N*_IG_ = 52); filled and unfilled circles indicate, respectively, EG (*N* = 188) and IG (*N* = 177) groups. The black curve shows the line of best fit from a linear-quadratic regression model, with 95% confidence intervals represented by the strip surrounded by black dashed lines. The marginal histograms on the top show the distributions of SC scores for each NAM (color) and group (solid and dashed traces). SC was higher for EG relative to IG groups in participants who mastered only 1 or 2 activities (NAM1 and NAM2), and was equivalent, with intermediate values, for participants who mastered all 3 activities (NAM3; top histogram). Source data are provided as a Source Data file.

Plotting dwfPC versus SC reveals two important insights (Fig. 4). First, dwfPC has a strong inverted-U relationship with SC, suggesting that the best learning outcomes were associated with intermediate SC. An additive model of dwfPC that included both linear and quadratic SC-index terms (as well as control variables of initial performance and instruction) was superior to its counterpart with only a linear term, Δ_AIC_ = 11.775). The linear-quadratic model accounted for a significant fraction of variance (${R}_{{{{{{{{\rm{adjusted}}}}}}}}}^{2}=0.159,\,F(4,360)=18.238,\,p \, < \, 0.001$) and produced a significant negative coefficient for the quadratic term (−0.016, *t*(360) = −1.966, *p* < 0.001). We replicated this finding when we repeated the analysis using unweighted final PC scores (${R}_{{{{{{{{\rm{adjusted}}}}}}}}}^{2}=0.191,\,F(4,360)=13.642,\,p \, < \, 0.001$, with the coefficient for the quadratic term = −0.017, *t*(360) = −3.561, *p* = 0.007) and when we replaced SC with pairwise contrast of activity choices (Supplementary Fig. 4b) showing that the finding was not an artefact of the specific ways we measured PC or SC.

Second, participants with different instructions and learning achievement fell on different portions of the inverted-U curve. Participants who did not master all 3 activities (NAM1 and NAM2) fell on the rising and falling arms of the inverted-U curve if they were in, respectively, the IG or the EG group (Fig. 4). These participants had equivalent dwfPC but higher SC in the EG relative to the IG group (multiplicative linear model; NAM1, *t*(359) = 2.856, *p* = 0.005; NAM2 (*t*(359) = 4.377, *p* < 0.001; Tukey’s HSD; see the marginal histograms in Fig. 4). Thus, EG participants who failed to master all 3 tasks did so because they over-challenged themselves and those in the IG group did so because they under-challenged themselves. In contrast, participants who mastered all 3 activities were at the top of the inverted-U curve and had equivalent (intermediate) SC in the IG and EG groups (Fig. 3c; no significant pairwise contrasts between EG and IG for NAM3, *t*(359) = 1.236, *p* = 0.217; see the top marginal histogram). Thus, consistent with the activity preferences (Fig. 3c): a subset of participants spontaneously adopted intermediate self-challenge strategies and maximized learning regardless of external instructions.

### Computational modeling and sensitivity to LP

While empirical studies demonstrate preferences for activities of intermediate complexity, they have yet to report specific sensitivity to LP. One study^38^ reports that people choose study words that are judged to have intermediate difficulty, but did not measure dynamic sensitivity to LP - the change in performance over time - either alone or in combination with PC.

To examine this question, we fit the participants’ activity choices by leveraging the formalism of intrinsically motivated reinforcement learning models^13,27,29,39^. Such models typically include three major components: (1) a space of learning activities, (2) an intrinsic utility function for each activity, associated with a decision-making mechanism, modeling how they are sampled, and (3) a model of learning mechanisms that improve skills after practicing an activity. Here, we already know the space of learning activities and we can observe the evolution of performance as learners engage in the activities. Thus, we can ask which intrinsic utility function could best explain the participants’ choices. To do so, we consider a standard softmax model (in a bandit setting^39^), in which the utility of an activity is a linear combination of PC and LP:

3.1 $${U}_{i,t}={w}_{{{{{{{{\rm{PC}}}}}}}}}\times {{{{{{{{\rm{PC}}}}}}}}}_{i,t}+{w}_{{{{{{{{\rm{LP}}}}}}}}}\times {{{{{{{{\rm{LP}}}}}}}}}_{i,t}$$

PC and LP were dynamically evaluated for each activity *i* at each trial *t* based on the recent feedback history. PC was defined as the number of correct guesses over the last 15 trials of activity *i*, and LP was defined as the difference in PC between first versus second parts of the same interval (similar to models of PC and LP used in refs. ^29,31,39^). We fitted each participants’ data (excluding 8 EG and 9 IG participants who did not master even a single activity) as a probabilistic (softmax) choice over 4 discrete classes, using maximum likelihood estimation with 3 free parameters - the softmax temperature (capturing choice stochasticity) and weights *w*PC, *w*LP indicating the extent to which each participant was sensitive to, respectively, PC and LP (Methods, Computational modeling). Supplementary Fig. 5 illustrates the model fitting procedure for an example participant’s data.

**Fig. 5:**
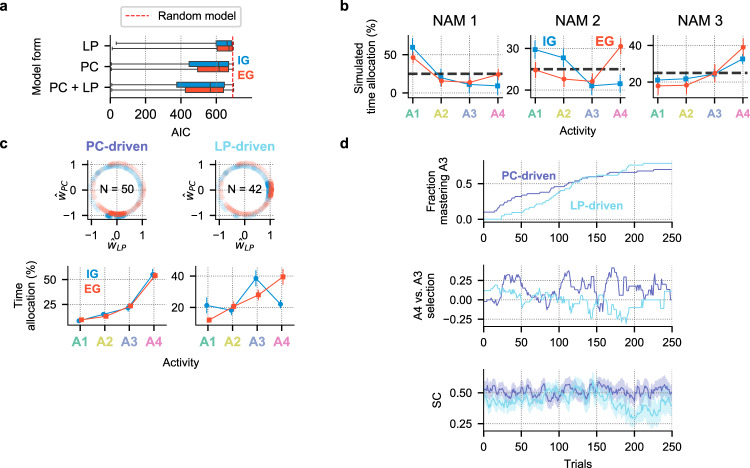
Computational modeling results. **a** The bivariate models had better AIC scores both across and within groups (*N*_EG_ = 188; *N*_IG_ = 177), compared to random-choice and univariate baselines univariate models. Box boundaries represent the 1st and the 3rd quartiles, and the lines inside show median scores; whiskers represent the full sample range. The dotted red line shows the AIC of the random-choice model. **b** Fitted coefficients reproduce choice patterns across instruction and NAM groups. The panels show the average time allocation patterns obtained by simulating activity choices over 250 trials using *N* = 500 randomly sampled coefficients from the pool of all fitted bivariate models. **c** Models of two distinct activity-selection strategies. The top row shows the joint distributions of normalized bivariate-utility coefficients. Subsets of these distributions whose data is presented below are highlighted with solid colors. These subsets were formed by first grouping all fitted models into three segments along $\hat{w}{{{{{{{\rm{PC}}}}}}}}$ and $\hat{w}{{{{{{{\rm{LP}}}}}}}}$, and then selecting groups corresponding to PC-driven and LP-driven profiles. Sample sizes of each subset are shown their respective subpanels. The bottom row shows mean relative frequencies of selecting each activity in the corresponding subset of participants depicted immediately above. LP-driven participants sampled the unlearnable activity (A4) in relative moderation compared the PC-driven group. **d** LP-driven participants selected allocated time more efficiently for learning and had better learning outcomes. The top row shows fractions of participants in the two groups that reached an objective criterion of 13/15 trials on the hardest learnable activity (A3) at least once in the experiment. The middle row shows the relative preference for activity A4 over A3, defined as the difference between fractions of participants (that still have not mastered A3) who selected A4 minus the fraction selecting A3. The bottom row shows average SC scores in the two groups (shaded regions indicate the standard error). Source data are provided as a Source Data file.

The bivariate form of the model that included both LP and PC (Eq. (3.1)) provided a superior fit to the data in both EG and IG groups. The bivariate model average AIC score (M = 491.992, SD = 200.389)) was lower than that of an alternative model based on random selection (M = 693.147; SD = 0; the baseline model yields the same likelihood regardless of participants’ choices; see Methods, Computational modeling, Eq. (5.5)) and, importantly, also outperformed univariate models that included only LP or only PC terms (Fig. 5a). A 2-way ANOVA of AIC scores showed a significant effect of model form (*F*(2, 1089) = 43.992, *p* < 0.001), a marginal effect of instruction (*p* = 0.054), but no interaction between model form and EG/IG groups (*p* = 0.716). The bivariate model had the lowest AIC scores in a large majority of participants in both groups (EG: 70.74%; IG: 74.01%). Finally, in each group, the bivariate model had a significantly lower AIC relative to each participant’s next-best model (Wilcoxon signed-rank test, EG: mean difference = 21.503, SD = 41.433; *Z*(188) = 55, *p* < 0.001; IG: mean difference = 21.882, SD = 45.383; *Z*(177) = 46, *p* < 0.001) and was at least 2 AIC points away from the next-best model in a majority of participants (EG: 58.51%; IG: 62.71%).

The fact that the bivariate model fits free-choice data better than univariate models provides direct evidence that participants are sensitive to LP – a heuristic for the temporal derivative of PC – above and beyond overall error rates. Importantly, the lack of interaction between model form and instruction shows that participants do not need to be explicitly instructed to maximize learning to demonstrate sensitivity to LP. Additional analyses showed that the PC and LP coefficients remained important even after including a term representing task familiarity (the reciprocal of novelty) in the utility function (see Supplementary Fig. 6). As discussed for Fig. S6, we focus on models without the familiarity term because in our task, novelty/familiarity is defined only by past choices and is thus circular if used to model choices. Modeling familiarity accounts for choice autocorrelation, but does not explain it. We note, however, that in computational RL studies^24,40^), measures of competence (like our PC) are used as a proxy for novelty preference that guides agents towards unfamiliar states.

As a final validation of our models, we conducted model simulations of time-allocation using the coefficients fitted by the bivariate models. We simulated activity choices over 250 trials in each NAM and EG/IG group using the observed success rates in conjunction with the fitted coefficients (randomly sampled with replacement over 500 iterations). As shown in Fig. 5b, the simulations reproduced the main patterns of time allocation, including the preference for activity A4 in the EG and IG NAM3 groups, and the preference for activity A1 in the NAM1 and NAM2 IG groups (see Fig. 3c for comparison), confirming that the bivariate models captured the main features of the empirical data.

Computational theories suggest that sensitivities to PC and LP will have distinct contributions to activity choices and learning. While a sensitivity to PC can motivate people to learn by steering them away from overly easy activities, a sensitivity to LP may protect them from focusing on overly difficult or impossible activities. Several aspects of the *w*PC and *w*LP coefficients in our task support these hypotheses.

First, the *w*PC and *w*LP were uncorrelated and showed different effects of instructions, suggesting that they capture different influences on choice strategies. We found no correlation between the *w*PC and *w*LP coefficients in the IG group (Pearson correlation of normalized coefficients, IG group: *r*(186) = −0.077, *p* = 0.298); EG group: *r*(175) = 0.062, *p* = 0.399; the normalization procedure is described in Methods, Computational modeling). Moreover, the PC coefficients were on average positive in the IG group and negative in the EG group (consistent with the groups’ relative preferences for easier versus harder activities) while the LP coefficients showed no effects of instructions (mean normalized PC coefficient in IG: M_norm_ = 0.255, SD = 0.724; in EG: M_norm_ = −0.232, SD = 0.741; 1-way ANOVA, *F*(1, 363) = 40.240, *p* < 0.001; mean normalized LP coefficient in IG: M_norm_ = 0.079, SD = 0.640; in EG: M_norm_ = 0.062, SD = 0.631; 1-way ANOVA, *F*(1, 363) = 0.065, *p* = 0.799).

Additional analyses supported the view that while both PC and LP coefficients correlate with higher self-challenge (Supplementary Fig. 4c), a sensitivity to LP can steer people away from unlearnable activities. We first conducted a group-level analysis of the correlation between the coefficients and two model-free measures of task choices: the difference between the time devoted to A3 versus easier activities (indexing the tendency to choose more challenging learnable activities) and the difference between the time devoted to activity A4 relative to the other activities (indexing the tendency to choose the unlearnable activity). Across all participants, lower PC coefficients coincided with a preference for choosing both A3 and A4, but $\hat{w}{{{{{{{\rm{LP}}}}}}}}$ coefficients correlated only with a preference for the learnable, A3 activity (Supplementary Fig. 7).

To more closely examine the specific contribution of LP sensitivity we focused on two subsets of participants whose choices were driven predominantly by, respectively, PC or LP. As shown in Fig. 5 (c, top), PC-driven participants had negative PC coefficients but near-zero LP coefficients and LP-driven participants had positive LP coefficients but near-zero PC coefficients (see Methods, Computational modeling for details of the grouping procedure). While both groups preferred more difficult activities (cf. Supplementary Fig. 4c) the preference for A4 was lower in LP-driven relative to PC-driven participants. Linear regression models of time allocation as a function of activity (A3 or A4) and type of drive showed that PC-driven people engaged in activity A4 more often relative to A3 in both the EG and IG groups (EG: slope = 76.485, *t*(104) = 7.019, *p* < 0.001; IG: slope = 83.941, *t*(72) = 5.199, *p* < 0.001) but this preference was lower or absent in LP-driven participants as shown by its negative interaction with the type of drive (EG: interactionslope = −47.628, *t*(104) = −2.726, *p* < 0.001; IG: interactionslope = −125.179, *t*(72) = − 5.764, *p* < 0.001).

Importantly, the lower preference for A4 enhanced learning outcomes in the LP-driven relative to the PC-driven group. As shown in Fig. 5d, after approximately trial 80, PC-driven participants showed a prominent increase in choices of A4 in favor of A3 but this was not seen in the LP-driven participants (Fig. 5d, middle row, captured as a decline in SC in the latter group (Fig. 5d, bottom row). At around the same time, the fraction of participants mastering A3 in the LP-driven group exceeded that in the PC-driven group (Fig. 5d, top row). By the end of the free-play stage, the probability of mastering at least 2 activities was 90.48% in the LP-driven group versus 70.59% the PC-driven group, and the probability of mastering all 3 tasks was, respectively, 64.29% versus 34.98%. Thus, consistent with theoretical predictions, LP-driven choices increase the efficiency of active learning by steering participants away from unlearnable activities.

## Discussion

While the ability to self-organize study time is critical for learning success, finding an efficient organization poses a daunting computational challenge. Prominent theories such as the free energy principle postulate that animals are intrinsically motivated to optimize their explanatory models of the environment^10,41^. However, the strategies for optimal exploration that are proposed by these theories are limited to highly simplified laboratory conditions while being typically too complex to be computed in real-world situations^42^. Similarly, mathematical models prescribing how students should allocate study time across competing activities show that optimal allocation is strongly sensitive to the precise shape of the learning trajectory, but this shape is typically unknown to the learner in advance^12^.

LP-based algorithms solve this conundrum by generating intrinsic rewards for activities in which learning recently occurred in practice, and thus provide a uniquely powerful means to optimize choices of study activity using a biologically plausible mechanism. And yet, it is unknown whether or how such choice strategies influence human behavior. Here we use a free-choice paradigm in which participants allocate study time based on dynamic feedback history and provide direct empirical evidence that humans are sensitive to LP.

Converging evidence suggests that humans tend to choose activities of intermediate complexity in a range of disparate settings - e.g., when spontaneously allocating visual attention in infancy (^19^, solving complex cognitive tasks) or declaring esthetic preference^20,21,43^. Our present results show that the preference for intermediate complexity extends to choices of learning activities (see also ref. ^18^) and, most importantly, that it may be a manifestation of an underlying LP-based mechanism. Thus, the ubiquitous preference for intermediate complexity reported in different settings may reflect an underlying mechanism that steers organisms toward activities that provide learning maximization.

Two major ideas in the literature postulate that exploration is structured based on the learner’s competence (prediction errors or error rates) or, alternatively, based on changes in competence over time (learning progress). However, whereas these strategies are typically framed as mutually exclusive alternatives^25,44–46^ our findings suggest that these two factors are uncorrelated and can jointly shape activity choices and contribute to different aspects of an investigative policy. A sensitivity to PC – with a preference for higher error rates – motivates people to explore more difficult unfamiliar activities, while a sensitivity to LP - the temporal derivative of PC - allows people to avoid unlearnable activities.

The properties of PC- and LP-based control mechanisms in our data suggests that the relative influence of each type of control may depend on the set of available learning activities. Here we used a relatively simple setting in which the available activities can be quickly mastered, and found that a PC-based strategy strongly contributed to the drive to choose challenging activities rather than stick with already-mastered tasks. However, if the environment is replete with challenging and unlearnable tasks, e.g., during realistic scientific investigation, an LP-based strategy may be more critical for steering learners toward tasks where progress is made as proposed in artificial curiosity^25,26,29^.

Our results also pertain to the relation between extrinsic and intrinsic motivation - and specifically the debate whether extrinsic rewards bolster^3^ or suppress^47^ the intrinsic motivation to learn. Our findings suggest that the answer is more complex, as external objectives both enhanced and impaired different aspects of our learners’ study strategy. On one hand, external objectives motivated participants to greater self-challenge, as people who were told to study for a test showed a greater tolerance for errors and better learning outcomes than those who did not. On the other hand, external instructions dampened learning achievement by inducing some participants to labor in vain on a random activity rather than learnable activity.

It is important to note that, while previous studies pitted intrinsic motivation against extrinsic monetary incentives (e.g.^47^), the extrinsic motivation for the EG group in our task came from the specification of a learning objective. In addition, rather than rewarding participants for individual correct answers, our external instruction specified the end-goal but not the local strategy for achieving the goal; this allowed people to choose their activities and commit errors in the short term, in the interest of maximizing learning in the long term. This greater autonomy, we believe, contributed to the synergism we observed, whereby externally imposed goals enhanced the eventual learning outcomes, rather than hindering them. Our findings support two key postulates of self-determination theory stating that intrinsic and extrinsic motivations are not dichotomous but fall on a continuum, and that a sense of agency is a strong factor that motivates people to internalize and meet externally imposed goals^48^. Thus, the most critical question may not be whether external objectives have beneficial or detrimental effects - but how to balance these objectives to support the investigative strategy that is most efficient in a particular context.

Last but not least, by examining investigations on longer time-scales, our results bear on the increasingly recognized distinction between momentary curiosity and sustained learning and interest^9,49^. Beyond the brief satisfaction offered by fleeting (diversive) curiosity, long-term sustained interest, and the willingness to exert sustained effort in pursuit of such interests, can have profound influence on the lifelong acquisition of competence and skills^9,50^. Hidi and Renninger^9,50^ proposed a four-stage model of interest development, whereby situational interests is initially triggered and sustained (or dampened) by the environment but with time gives way to well-developed interest in which people spontaneously generate new questions and initiate investigations^38^. The fact that many people in our IG group mastered two or more tasks and reported subjective interest proportional to their time allocation (Supplementary Fig. 2), suggests that the activities we provided may have triggered their situational interest regardless of explicit instructions. The fact that higher achievements were more common in the EG group suggests that external instructions help support that fledgling interest. Thus, important questions for future research concern the relation between the mechanisms by which people self-organize their activities, their subjective feelings of interest and the impact of both factors on the development of lifelong interests and skills.

Finally, the experimental setup implemented in our study allows researchers to fit and evaluate a larger scope of models. In this study we focused exclusively on modeling activity choices while eschewing assumptions about the learning process itself and the potentially complex factors that modulate it (including, e.g., forgetting, switching costs, effort, and preferences for uncertainty). However, follow up work can easily extend the task design to allow for proper examination of these factors, for example by collecting subjective probability ratings to track participants’ evolving inferences regarding each task. Moreover, while our task takes a step towards a more naturalistic lab setting by giving people the freedom to choose their own learning activities it supplies a very limited set of learning activities. Future studies can benefit from the straightforward parametrization of the learning environment (e.g., number of learning activities, difficulty levels, number of response categories, time horizon, etc.) to study how different drives self-organized learning according to context.

## Methods

Four-hundred participants (including 208 female, 187 male, and 5 participants of undisclosed gender) were recruited for the study on the online platform Amazon Mechanical Turk. Participants were between 19 and 71 years of age, with an average age of 36.15 years, SD = 10.54). All participants provided informed consent. All the procedures were approved by the Institutional Review Board of the University of Rochester.

All participants were told that the experiment will last 45 min to 1 h and, upon completion, they will be compensated $1 regardless of performance. This scheme was consistent with prevailing rates on Amazon MTurk and with our goals of minimizing the role of monetary incentives and avoiding biasing participants toward activities with consistently high performance. All participants were asked to complete the task on their own in a quiet environment and eliminate external distractions (e.g., turn off cell phones, TV sets, music players, etc). After receiving detailed written instructions, each participant completed 4 task modules in sequence: (1) 15 forced-choice familiarization trials with each activity; (2) rating of prospective learnability for each task (see below); (3) a free-play stage with 250 trials of free-choice of activity; (4) 6 additional subjective ratings (see below).

Before delivery of the instruction, participants were randomly assigned to the EG and IG groups, who received identical treatments except for the initial instruction. The IG participants received a task description that did not communicate any expectations or objectives on the part of the experimenters: “In each family there are several individuals, and the appearance of an individual might predict what food they like to eat. When you interact with a monster family, different individuals will be presented to you. For each individual, two food items will be displayed, and you can click on the one you think it prefers. You will receive feedback whether your guess was correct or not”, which was followed by brief descriptions of familiarization, free-choice, and questionnaire stages. The EG participants’ instructions were identical, except for two additional sentences that included an explicit prescription of a learning goal: “In the main section of the task, we ask you to play for 250 trials and try to maximize your learning *about all the 4 families*” followed by information on the post-session testing module re-emphasizing their objective: “We will briefly test how well you learned to predict the food preferences within each family”. After the free-play stage, participants in the EG group received the announced test (between steps 3 and 4) consisting of 15 forced-choice trials on each activity. (However, in our analyses we used the last 15 trials on the free-play stage rather than the test data, as the latter were not available for the IG group). Participants in both groups also provided several ratings of the activities, described in detail in Supplementary Fig. 2.

### Data analysis

Statistical analyses were performed in using the R 3.5.0 (relevant libraries include contrast 0.22, emmeans 1.4, MASS 7.3.51.5, tydyverse 1.3.0 and rstatix 0.6.0). Data wrangling, data visualizations, and computational modeling were done in Python 3.6 (relevant libraries include matplotlib 3.2.2 and seaborn 0.11.0 for data visualizations; numpy 1.19.0, scipy 1.5.1, and pandas 0.24.1 for data wrangling, visualizations, and modeling). Complete lists of Python and R libraries and sub-dependencies is provided in the code repository (see Code availability). All the *t*-tests reported throughout this article and supplementary information are two-tailed. We excluded a total of 18 participants – 5 in the EG and 13 in the IG group – who did not appear to be sufficiently engaged in the task based on a response bias criterion (see Supplementary Fig. 1 for more details). This criterion measured the participants’ tendency to choose a single response category in each activity (i.e., always guessing the same food item, regardless of the stimulus).

#### Difficulty-weighted final performance

Difficulty weighted final PC (dwfPC) is a weighted average of each participant’s finalPC(fPC) on the learnable activities over the last 15 trials played on the activity. The weights are equal to the activity rank (1, 2 and 3) divided by the sum of the ranks (6). Thus, dwfPC for participant *i* is ${{{{{{{{\rm{dwfPC}}}}}}}}}_{i}=\frac{1}{6}{{{{{{{{\rm{fPC}}}}}}}}}_{i,A1}+\frac{1}{3}{{{{{{{{\rm{fPC}}}}}}}}}_{i,A2}+\frac{1}{2}{{{{{{{{\rm{fPC}}}}}}}}}_{i,A3}$. (Here and in all subsequent analyses we chose a 15-trial time window that was equal to the number of familiarization trials each participant played).

#### NAM designation

We divided participants into discrete groups based on the number of activities on which they reached a mastery criterion. The data presented in this article are based on a criterion of 13/15 correct trials (86.7% correct), which, in a binomial distribution with discrete outcomes, corresponds to *p* = 0.0037 of arising by chance. Additional analyses verified that the conclusions are robust over a range of criteria (see Supplementary Fig. 3). Ten participants (5/154 in the IG group and 5/176 in the EG group) did not master any activity and were excluded from NAM-related analyses and computational modeling.

#### Self-challenge index

For each participant, we defined a self-challenge (SC) index for each trial *t* and activity *i* as:

5.1 $${{{{{{{{\rm{SC}}}}}}}}}_{t,i}=1-\frac{{{{{{{{{\rm{PC}}}}}}}}}_{t,i}-\mathop{\min }\limits_{\forall k\in K}{{{{{{{{\rm{PC}}}}}}}}}_{:t,k}}{\mathop{\max }\limits_{\forall k\in K}{{{{{{{{\rm{PC}}}}}}}}}_{:t,k}-\mathop{\min }\limits_{\forall k\in K}{{{{{{{{\rm{PC}}}}}}}}}_{:t,k}}$$

where PC_*t*,*i*_ is the recent PC of the selected activity the participant selected on trial *t* (measured over the last 15 trials on that activity, including familiarization trials) and where ${\min }_{\forall k\in K}{{{{{{{{\rm{PC}}}}}}}}}_{:t,k}$ and ${\max }_{\forall k\in K}{{{{{{{{\rm{PC}}}}}}}}}_{:t,k}$ are the minimum and maximum PC experienced by the participant over the entire set of trials (including both free- and forced choice) prior to trial *t* and over the entire set of activities *K*. Thus, SC values close to 1 indicate a tendency to select activities that yield the minimum PC (“over-challenging”) and values closer to 0 indicate a tendency to select activities with the highest PC (“under-challenging”). To get a single SC index for each participant, we averaged each participants’ the trial-wise SC scores across the entire free-play stage. Supplementary analyses verified that the SC index was a better, more concise measure of the preference for challenging tasks relative to the pairwise preferences between different combinations of activities (see Supplementary Fig. 4).

#### Computational modeling

To understand which intrinsic utility function could best explain the task sampling behavior, we consider a model in the bandit setting (^39^), where an intrinsic utility function for each task, measuring its value, is used to decide which task to sample probabilistically. The sampling mechanism used here is the softmax function, following prior models of human decision making in RL and bandit settings^51^. This softmax function simultaneously translates the underlying choice utilities into selection probabilities and scales the correspondence between utility and probability:

5.2 $${p}_{t}({{{{{{{{\rm{choice}}}}}}}}}_{i})=\frac{{e}^{{U}_{i,t}\times \tau }}{{\sum }_{\forall k\in K}{e}^{{U}_{k,t}\times \tau }}$$

*U*_*i*_ is the subjective value of choice *i*, and *k* indexes the utilities of all items in the set of available activities *K* (including *i*); the parameter *τ*, known as temperature, controls how strongly the item values determine the probability of their selection. *U* was defined for each trial as described in the Results section (Computational modeling and sensitivity to LP), as a linear combination of two quantities that represent two aspects of learning: competence and change in competence. Both signals were defined for a retrospective time window of the last 15 trials played on the activity chosen at trial *i* (including familiarization trials early in the free-play epoch):

5.3 $${{{{{{{{\rm{PC}}}}}}}}}_{i,t}=\frac{1}{15}\mathop{\sum }\limits_{t^{\prime} =t-15}^{t}{y}_{t^{\prime} }$$

5.4 $${{{{{{{{\rm{LP}}}}}}}}}_{i,t}=\left|\left(\frac{1}{10}\mathop{\sum }\limits_{t^{\prime} =t-15}^{t-5}{y}_{t^{\prime} }\right)-\left(\frac{1}{9}\mathop{\sum }\limits_{t^{\prime} =t-9}^{t}{y}_{t^{\prime} }\right)\right|$$

where ${y}_{t^{\prime} }$ equals 1 or 0 if the participant guessed, respectively, correctly or in error at time $t^{\prime}$. Hence, PC was defined as the proportion of correct guesses over the last 15 trials, while LP was defined as the absolute value of the difference in PC over the first 10 and the last 9 of the same stretch of 15 trials. This implementation of PC and LP signals is similar to machine learning models in refs. ^29,31,39^. In particular, one follows these computational approaches in using the absolute value of LP, which was shown to enable learners to detect tasks where performances decrease, e.g., due to forgetting, and re-gain interest to re-focus on them^29^.

An individual set of parameters was estimated for each participant by minimizing the negative sum of log likelihood values over the free play trials (see ref. ^52^). Assuming that choice probabilities on a trial come from a categorical probability distribution, the likelihood of a model equals the probability (provided by the model) of the observed choice. The categorical distribution is a special case of the multinomial probability distribution, which provides the probabilities of *K* discrete outcomes in a single sample. Thus, the likelihood of a model that predicts choices with probabilities *p*_*t*_ is:

5.5 $$L({{{{{{{{\bf{p}}}}}}}}}_{t}| {{{{{{{{\rm{choice}}}}}}}}}_{i})=f({{{{{{{{\rm{choice}}}}}}}}}_{i}| {{{{{{{{\bf{p}}}}}}}}}_{t})=\mathop{\prod }\limits_{j=1}^{K}{p}_{t}{({{{{{{{{\rm{choice}}}}}}}}}_{j})}^{[i = j]}$$

where **p**_*t*_ is a vector of probabilities at time *t* associated with *K* items indexed by *j*, and the term [*j* = *i*] evaluates to 1 when *i* is the activity that was chosen and to 0 otherwise. Thus, at the level of a single trial, higher likelihood is attributed to the model that assigns higher utility to the option chosen on the subsequent trial. For two and more trials, the likelihood of a model increases with the utility of the observed choices across trials. Therefore, in a maximum-likelihood model, a highly positive coefficient for a given learning signal reflects a tendency to choose options with higher values along that signal. Conversely, a highly negative coefficient for a feature indicates a tendency to choose options that have lower values along that feature, while coefficients close to zero reflect the indifference to the feature. The total likelihood of observing all choices from a participant is given by the product of likelihoods from individual trials, $\mathop{\prod }\nolimits_{t}^{T}L({{{{{{{{\bf{p}}}}}}}}}_{t}| {{{{{{{\rm{choice}}}}}}}})$. We take a logarithm of each individual trial’s likelihood value in order to compute the overall model likelihood per individual as the sum of single-trial log likelihoods, $\mathop{\sum }\nolimits_{t}^{T}{{{{{{\mathrm{log}}}}}}}\,L({{{{{{{{\bf{p}}}}}}}}}_{t}| {{{{{{{\rm{choice}}}}}}}})$, rather than their product. Finally, we maximized this summed likelihood by minimizing its negative value using the L-BFGS-B nonlinear numerical optimization method^53^.

Values of the estimated parameters vary not only due to different choice data between participants, but also as a function of initialization of starting values in the parameter space. Because of this variability, we estimated a model multiple times for each participant using different parameter initializations for every fit, until a convergence criterion was reached. The utility parameters were initialized from a random uniform distribution between −1 and 1, and softmax temperature was randomly sampled from [0, 100]. Convergence was reached by repeatedly fitting a model with different random initializations until 50 maximum likelihood models were found. Concretely, the algorithm updated the current "best model" each time a model better the current best was found, and stopped when it found a model just as good as the current best 50 times.

For analyses of the relation between the coefficients, instructions and choices, we normalized each coefficient pair [*w*PC, *w*LP] by their Euclidean norm, allowing us to interpret the coefficients as relative preferences for PC and LP, respectively.

To select participants driven predominantly by PC or LP (Fig. 5c, d), we categorized all participants into equally-spaced bins (bin_1_ = [−1.00, −0.33); bin_2_ = [−0.33, 0.33); bin_3_ = [0.33, 1.00]) along each (normalized) coefficient. The PC-driven group (Fig. 5c, left) had negative PC coefficients but near-zero influence of LP (intersection of bin_1_ along PC and bin_2_ along LP i.e., ${\hat{w}}_{{{{{{{{\rm{LP}}}}}}}}}\approx 0,\,{\hat{w}}_{{{{{{{{\rm{PC}}}}}}}}}\approx -1$), while the LP-driven group (Fig. 5c, right) had a high preference for LP but little preference to PC (${\hat{w}}_{{{{{{{{\rm{LP}}}}}}}}}\approx 1,\,{\hat{w}}_{{{{{{{{\rm{PC}}}}}}}}}\approx 0$).

### Reporting summary

Further information on research design is available in the Nature Research Reporting Summary linked to this article.

## Supplementary information

Supplementary Information

Description of Additional Supplementary Files

Supplementary Software 1

Reporting Summary

## Data Availability

The datasets generated and/or analyzed during the current study are available in the Open Science Framework public repository, https://osf.io/k2yur/, which includes datasets derived from raw data as well as the raw data themselves. All source data used for visualizations and analyses are also provided with this paper as a Source Data file. Source data are provided with this paper.

## Source data

Source Data

## Code availability

We share all the code used in quantitative analyses (including visualizations) and computation modeling on GitHub, https://github.com/flowersteam/Humans-monitor-LP. A permanent version of the code repository for the figures and analyses is archived on Zenodo 10.5281/zenodo.5179939. All source code usef for visualizations and analyses is also provided with this paper as a Source Code file.
