# Supplementary material for: Humans monitor learning progress in curiosity-driven exploration
Source: Nat Commun. 2021 Oct 13;12:5972. doi: 10.1038/s41467-021-26196-w (PMC8514490; doi:10.1038/s41467-021-26196-w)
Supplement: Supplementary file 1 — Supplementary Information [file 41467_2021_26196_MOESM1_ESM.pdf]

Humans monitor learning progress in curiosity-driven exploration (Supplementary Information)

Alexandr Ten*[1], Pramod Kaushik[1], Pierre-Yves Oudeyer[1], and Jacqueline Gottlieb[2]

[1]INRIA Bordeaux Sud-Ouest, 200 Avenue de la Vieille Tour, 33405 Talence, France
[2]Department of Neuroscience & The Kavli Institute for Brain Science, Columbia University, 1051 Riverside Drive, Kolb Research Annex, Rm. 569, New York, NY 10032, United States
*alexandr.ten@inria.fr

This document provides supplementary information for the quantitative analyses reported in the main article. All t-tests reported in this document are two-sided. The supplementary information is organized into 7 separate sections:

1. Exclusion Criteria provides the details of our data exclusion rationale.
2. Self-Reported Ratings provides supplementary analyses of some of the collected subjective judgments in order to evaluate participants' engagement in our task.
3. Mastery Points discusses the effects of using an objective performance criterion on the related analyses (specifically, analyses invoking the notion of "mastering" an activity)
4. Self-Challenge Index provides an extended discussion of the measure of self-challenge.
5. Individual Model Fit: A Case Study illustrates our procedure for the computational model fitting, using data from a single participant as concrete example.
6. Familiarity Component provides a more in-depth treatment of the possibility of including a familiarity component in the utility-based choice model.
7. Model Coefficients and Activity Preferences provides an additional analysis involving the coefficients from our computational modeling (specifically, relating the fitted model coefficients and behavioral preferences for harder activities)

1 Exclusion Criteria

We excluded 15 participants (11 in EG and 4 in IG group) based on a response bias criterion that characterized the level of engagement in the task. Response bias was defined as:

$$\text{response bias} = \frac{1}{K}\sum_{k=1}^{K}\max(p_k, 1-p_k) \tag{1.1}$$

where $K = 4$ is the number of learning activities and $max(p_k, 1 - p_k)$ denotes the relative frequency of the more frequently chosen response category in activity k. It corresponds to the participant's tendency to choose one kind of response across all trials.

Supplementary Fig. 1 shows the joint distribution of response bias scores in our sample, grouped by instruction. The figure also shows the excluded participants and the exclusion criterion. The vast majority of participants were below 0.7 which corresponded to 2 standard deviations above the mean. Relative to the included participants, the excluded ones had significantly shorter reaction times to choose a category (M = 1023.89, SD = 720.770 vs M = 1472.44, SD = 360.020; $t(23.99) = -4.484$, $p < .01$, Welch two-sample test) and significantly lower difficulty-weighted final percent-correct scores (dwfPC; M = .689, SD = .090 vs M = .704, SD = .080; Welch two-sample test, $t(19.93) = -2.361$, $p = .029$), suggesting that they responded in a stereotyped fashion without being engaged in the task.

2 Self-Reported Ratings

We collected self-reported ratings about all 4 activities at two different points of the task. Immediately after the familiarization stage (see Methods in the main article), participants were asked to report a single judgment of prospective learnability for each task:

- *Prospective learnability*: Before continuing, please rate each monster family based on how much you

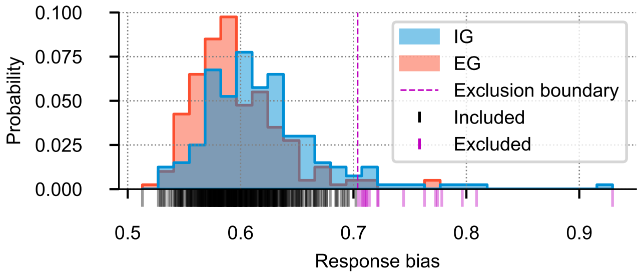

Supplementary Figure 1: Distribution of response bias scores ($N = 400$) used to exclude participants who were disengaged in performing the task. Vertical bars underneath the plot show individual data-points. The exclusion criterion, depicted as a vertical dashed line, was set to 2 standard deviations. Source data are provided as a Source Data file.

think you can learn about its food preferences during the rest of the task ([1] Definitely cannot learn more – [10] Definitely can learn more)

After responding to the first post-familiarization question, participants proceeded to play out the free-choice stage, after which we collected 6 additional ratings:

- *Interest*: Rate each monster family based on how much you were interested in discovering what they preferred eating ([1] Less interested – [10] More interested)
- *Complexity*: Rate each monster family based on how complex you thought they were ([1] Less complex – [10] More complex)
- *Rule*: Rate each monster family based on how likely you think it had a rule for food preferences ([1] Definitely no rule – [10] Definitely a rule)
- *Potential future learning*: Rate each monster family based on how much more you think you could learn if you had more time to play with it ([1] Definitely could not learn more – [10] Definitely could learn more)
- *Time spent*: Rate each monster family based on how much time you spent on them ([1] Less time – [10] More time)
- *Progress made*: Rate each monster family based on how much progress you felt you made for learning their food preferences ([1] Less progress – [10] More progress)

The subjective reports enabled us to assess how participants felt about various aspects of our task. Specifically, we were interested in two questions: (1) How well did participants track their performance and choices during free exploration? and (2) How interested were they in the activities?

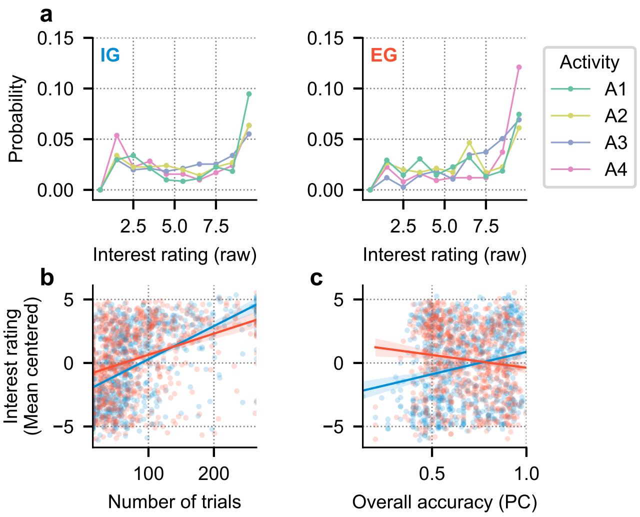

Supplementary Figure 2: **a**, Histograms of the raw retrospective interest ratings (1 to 10; collected after the free-play stage) for each activity in the IG (left; $N = 186$) and EG (right; $N = 196$) groups show that both groups had modes for the highest rating (10). **b**, Relationship between self-reported interest (y-axis) and number of trials for which an activity was chosen (x-axis). **c**, Relationship between self-reported interest (y-axis) and overall activity accuracy (x-axis). Note, in **b** and **c**, raw data points are presented for each instruction group (red for SG and blue for IG); regression lines are fitted separately for each group; error bands correspond to 95% confidence intervals for the linear predictions. Source data are provided as a Source Data file.

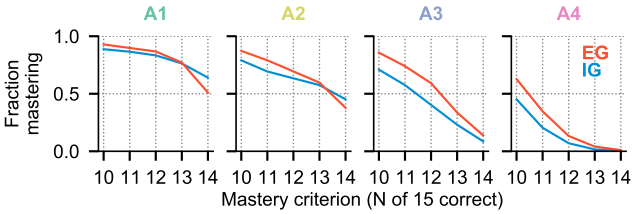

Supplementary Figure 3: Fractions of participants mastering each activity as a function of mastery criterion and group. Changing the criterion does not change the relative proportions of participants mastering each activity.

2.0.1 How well did participants track their performance and choices during free exploration?

Analyses of the progress ratings showed that participants had good awareness of their performance in both the EG and IG groups. Across participants and activities, self-reported Progress made was significantly correlated with true progress made (the difference between PC on the last 15 and first 15 trials on each activity; EG: $r(750) = .286$, $p < .001$; IG: $r(706) = .427$, $p < .001$). Similarly, self-reported Time spent was highly correlated with the true number of trials played (Pearson correlations, EG: $r(750) = .336$, $p < .001$; IG: $r(706) = .476$, $p < .001$). Thus, participants in both EG and IG groups accurately evaluated the relative time allocation and the progress they made across learning activities.

2.0.2 Were the participants interested while playing our task?

Although participants dutifully completed the requested 250 trials of the task, they could have, in principle, reported that they were not at all interested in the activities. Contrary to this view, the distribution of Interest ratings showed a strong peak at the highest rating (10) and the average ratings were above 5 even for the activities with the lowest average ratings in each group (A4 in IG: M = 5.371, SD = 3.432, and A1 in EG: M = 5.934, SD = 3.118; Supplementary Fig. 2, a).

Importantly, interest ratings scaled with the number of trials spent on each activity above and beyond the success rates (Supplementary Fig. 2, b). A linear regression model of mean-centered interest rating as a function of the total time spent on an activity (controlling for overall accuracy (PC over 250 trials) and the the instruction received, IG vs EG), showed that ratings were reliably predicted by the actual time spent in both the IG and EG groups (slope for IG group = 7.966, $t(1454) = 15.204$, $p < .001$; interaction slope = -2.941, $t(1454) = -3.957$, $p < 0.001$). Importantly this relation was independent of any effect of PC, suggesting that interest reflected more than mere success rates. Moreover, the correlation between PC and interest ratings was not significant in the IG group (slope = 0.379, $t(1454) = 0.646$, $p = .518$; Supplementary Fig. 2, b), and negative in the EG group (interaction slope = -2.941, $t(1454) = -3.957$, $p < .001$; Supplementary Fig. 2, b), suggesting that participants had an interest in the task that was above and beyond maximizing correct feedback.

Supplementary Table 1: Results of quadratic-regression fits of average SC on activity preference for each pairwise preference of a harder over easier activity. Source data are provided as a Source Data file.

		coef	t	p
A2 - A1	intercept	0.452	69.079	$< .01$
	pref	0.033	5.636	$< .01$
	pref^2	-0.060	-17.615	$< .01$
A3 - A1	intercept	0.426	59.860	$< .01$
	pref	0.078	12.060	$< .01$
	pref^2	-0.034	-8.470	$< .01$
A3 - A2	intercept	0.408	48.276	$< .01$
	pref	0.048	6.163	$< .01$
	pref^2	-0.016	-4.383	$< .01$
A4 - A1	intercept	0.397	62.827	$< .01$
	pref	0.126	23.322	$< .01$
	pref^2	-0.004	-1.096	$= .274$
A4 - A2	intercept	0.383	50.161	$< .01$
	pref	0.100	14.971	$< .01$
	pref^2	0.009	2.373	$= .019$
A4 - A3	intercept	0.372	44.348	$< .01$
	pref	0.058	7.736	$< .01$
	pref^2	0.02	5.232	$< .01$

Note: t-tests compare coefficient values against 0 (df = 362)

3 Mastery Points

The learning criterion we present in the main text was based on people achieving 13 of 15 consecutive correct trials on an activity, which is equivalent to PC = 86.7% and, in a binomial probability, has p = 0.0037 of occurring by chance. To ensure that our conclusions were robust to choice of criterion, we repeated the analyses with criteria of 10, 11, 12, 13, and 14 correct trials out of 15. As expected, the fraction of people mastering each task declined as the criterion increased but, critically, the relative frequencies of the NAM designations between EG and IG groups do not change (Supplementary Fig. 3). To test this, we performed a logistic regression of reaching the criterion (0 or 1) as a function of criterion and group (EG/IG). We performed a separate regression for each learning activity. We used repeated contrasts for the criterion factor to compare the fractions of participants mastering a task between the adjacent levels of the factor (i.e., comparing 10 vs 11, 11 vs 12, and so on), and regular treatment contrasts to compare fractions between groups.

The regressions produced no significant interactions between group and criterion (all $p > .05$) with only one exception: the mastery criterion of 14/15 correct was significantly less likely to be reached compared to 13/15 in the IG group (slope = -1.035, $Z(1909) = -4.155$, $p < .001$) and even less likely in the EG group (interaction slope = -0.802, $Z(1909) = -2.252$, $p < .024$). These results show that the differences between instruction groups were mostly stable over a range of criteria (as shown in Supplementary Fig. 3). The positive result for the 14/15 vs 13/15 contrast shows that exceptionally high performance (PC = 94%) was more likely to be reached on the easiest task and that EG participants seemed less interested in reaching this level of accuracy. Despite this effect, these mostly nonsignificant results show that an important observation holds across a range of mastery criteria: a significant fraction of the IG group achieved mastery without being instructed to maximize learning.

4 The Self-Challenge Index

We conducted several analyses that established that our SC measure captured the tendency to choose more difficult activities (Supplementary Fig. 4, a), did not bias our conclusions (Supplementary Fig. 4, a),and showed the expected correlations with the model coefficients (Supplementary Fig. 4, c).

As shown in Supplementary Fig. 4 (a), the SC index showed a positive correlation with all possible pairwise measures of the preference for the harder activities, confirming that it measured self-challenge. However, several of these relationships were non-linear, indicating that pairwise differences do not fully capture the choices in our 4-alternative task (see Supplementary Tab. 1 for full details on the regression fits). Specifically, the preferences for activities with moderate difficulty (A2 or A3) had an inverted U-shape trend indicating that, if these preferences were too strong they implied lower SC

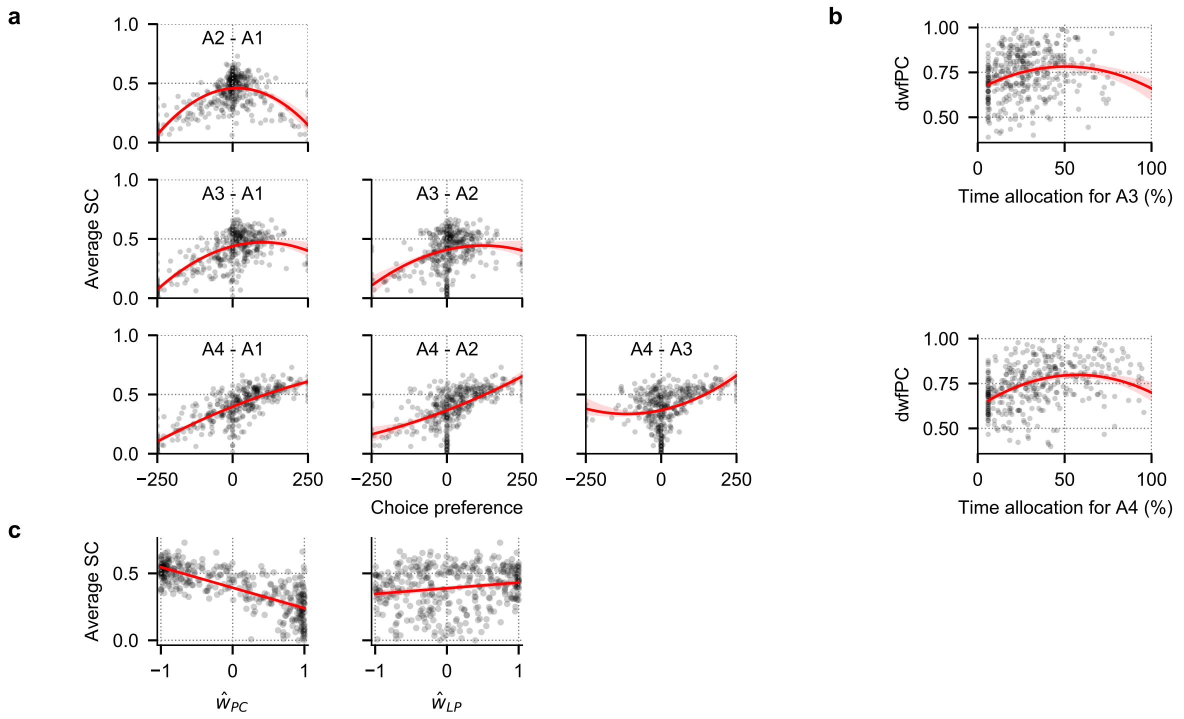

Supplementary Figure 4: **a**, Correlation between behavioral preferences for harder activities (x-axes) and average SC (y-axis). Each point indicates one participant (pooled across groups: EG, N = 188 and IG, N = 177). In each panel, the x-axis is constructed so that positive values show preference for the more difficult of the two contrasted activities. **b**, Correlation between time allocation scores (x-axis) and difficulty-weighted final performance (dwfPC; y-axis). **c**, Correlations between the normalized fitted coefficients (x-axes) and average SC (y-axis). In all the panels, red lines show fits of linear-quadratic regressions with error-bands (shaded regions) indicating 95% confidence intervals (details for the fits in a are in Supplementary Tab. 1). Source data are provided as a Source Data file.

by virtue of withdrawal from the most difficult activity. Similarly, the contrast of A4 vs A3 showed an upright U-shaped profile indicating that a lower preference for A4 can correspond with higher SC if people strongly prefer A3 over A1 and A2. Thus, in our 4-alternative choice experiment, the SC index is a more parsimonious measure of the preference for challenging tasks relative to measures of preference between specific pairs of tasks.

As additional confirmation, we verified that the inverted-U relationships between SC and dwfPC shown in the main text (4) was replicated if we replaced SC with the preference for A3 or A4 (Supplementary Fig. 4, b). In case of A3's time allocation, the linear-quadratic model was better than its non-quadratic counterpart (ΔAIC = 22.238) and showcased a significant negative coefficient for the quadratic term (slope = -0.016, $t(361) = -4.979$, $p < .001$). A similar linear-quadratic model featuring time allocation for activity A4 was also better than the corresponding non-quadratic model (ΔAIC = 26.178) and likewise had a significant coefficient for its quadratic term (slope = -0.026, $t(361) = -5.383$, $p < .001$). Together, the results from Supplementary Fig. 4, (a, b), demonstrate that SC served as a parsimonious measure of activity preferences and did not bias the results we report.

Finally, we examined how SC was related to the fitted (bivariate) computational-model coefficients (Supplementary Fig. 4, c). SC was negatively correlated with w_{PC} (slope = -0.153, $t(361) = -21.999$, $p < .001$), consistent with our intuitions that choosing activities with lower PC corresponds to self-challenging choices. The regression also showed a positive correlation with w_{LP} (slope = 0.042, $t(361) = 4.954$, $p < .001$), consistent with the prediction that a sensitivity to LP guides learners to venture beyond what's easy and familiar, and choose moderately challenging activities.

5 Individual Model Fit: A Case Study

Supplementary Fig. 5 demonstrates our model fitting procedure and model-based simulations for one participant's data. Panels a and b show, respectively, the participant's values of percent correct (PC) and learning progress (LP) values over time. PC and LP remained constant if the participant did not choose a task, explaining the long horizontal lines on the plots. Panel

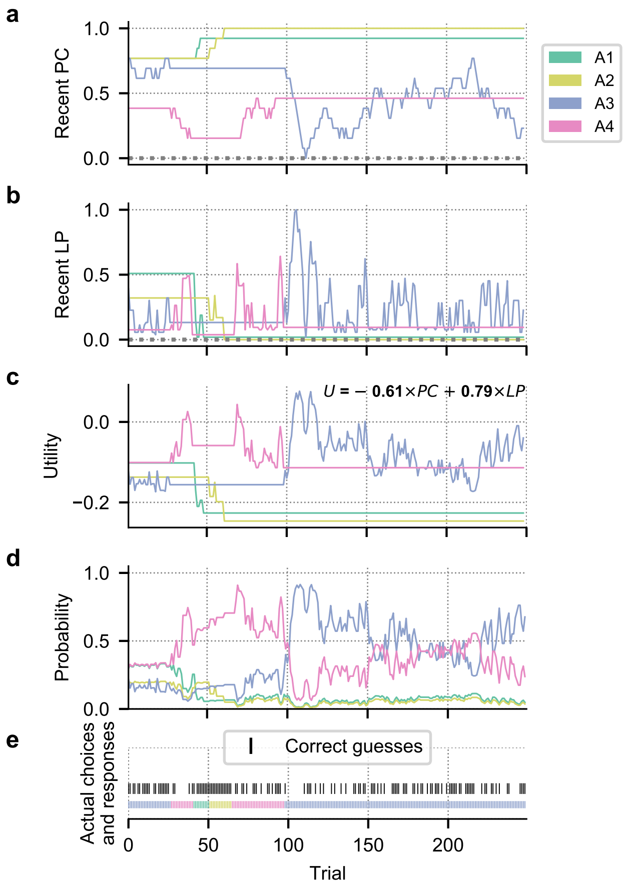

Supplementary Figure 5: The x-axis shows 250 trials of free play. Each of the first four subfigures shows the choice features of each activity through time. **a**, normalized recent percent correct (PC); b, normalized recent learning progress (LP); **c**, utility computed as a linear combination of PC and LP. The utility equation shows coefficients normalized by the Euclidean norm of the w_{PC} and w_{LP} coefficients; **d**, choice probabilities given by a softmax function at the fitted temperature parameter, $\tau = 84.98$; **e**, empirical data that the model was fitted to. The colored bar represents the observed sequences of activity choices (A3→A4→A1→A2→A4→A3) and the black vertical sticks show correct responses. Source data are provided as a Source Data file.

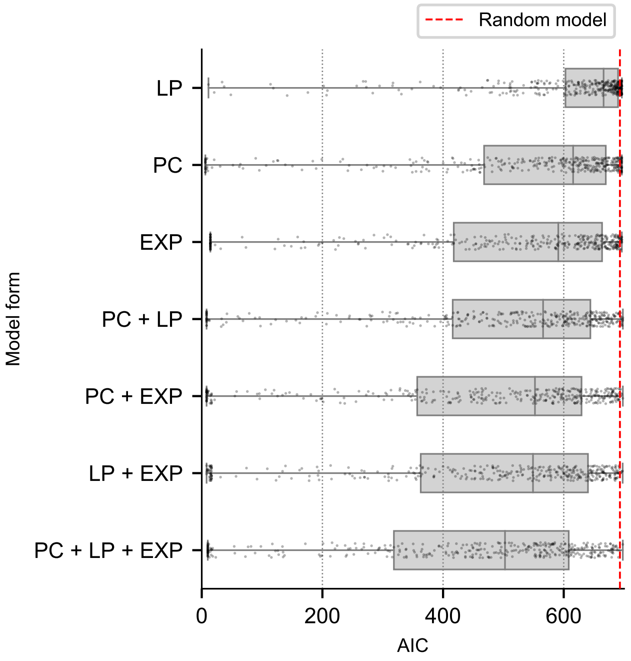

Supplementary Figure 6: Distributions of AIC scores for all model subsets of the full trivariate model PC + LP + EXP, where EXP represents count-based task familiarity. The box-plot box boundaries represent the 1st and the 3rd quartiles; middle bars represent sample medians; whiskers show sample minima and maxima. The random (baseline) model has AIC = 693.147 and no variance. Individual data points ($N = 365$ per model form) are shown in the overlaid strip-plots. Source data are provided as a Source Data file.

c shows the dynamic utility for each task based on the participant's fitted coefficients (given in the equation). Panel d shows the probabilities of choosing each task, simulated using the corresponding utility and the softmax function with the best-fit temperature parameter.

In this particular model, the participant's choices were characterized by a preference towards activities with high LP and low PC, which results in a model that predicted high probabilities of choosing A3 and A4 activities. Activities A1 and A2, which had consistently high PC values generated low utility and were infrequently chosen. This model captures well the transition from A4 to A3 around trial 100, where the utility of A4 started dropping as a result of a low LP signal and a corresponding plateauing of the PC signal (Supplementary Fig. 5, a, b, and d).

Note that we present a readily interpretable model fit and model predictions. The reader can explore visualizations of other models in a freely available Jupyter notebook (figures.ipynb) on a GitHub repository where we share all our code for modeling, statistical analyses, and visualizations (link: `https://github.com/flowersteam/Humans-monitor-LP`).

6 Familiarity Component

The model comparisons from the main text show that on average the bivariate utility function (PC + LP) explains participant's choices better than the univariate models. The measures of PC and LP capture different aspects of competence that were hypothesized to function as intrinsic reward signals for a freely exploring learner. Different analogs of these measures have been widely used in the computational literature on intrinsically motivated learning, e.g., [1, 2]. These measures can be characterized as competence-based measures, because they track the information about one's competence in performing a task. There are other important families of approaches which we did not include in our study. For instance, we did not include any predictive knowledge-based measures which would require to explicitly model the participants' beliefs about food preferences. This is an interesting direction for future work, but these kinds of models entail considerable additional complexity that is outside the scope of our investigation. However, we could test another kind of knowledge-based curiosity measure which does not require an internal predictive model. In computational literature, this approach is referred to as a count-based, because it relies on state visitation counts [3]. State visitation counts can be interpreted as state familiarity (the opposite of novelty).

Due to the reasoning laid out below, we did not include the measure of familiarity in the main report of model comparisons, even though it is a central idea behind some approaches to intrinsically motivated exploration. The rationale for omitting this component from the reported analyses was our focus on the roles of learning-based heuristics in the self-determined selective engagement in one of several learning activities. The familiarity measure, as defined below, is based directly on the participant's choices and as such is completely orthogonal to the dynamics of a learner's competence. Since a single episodic sampling of a learning activity contributes to the measured familiarity of all activities equally, familiarity measured this way does not merely correlate with activity choices, it is completely determined by them. Such a

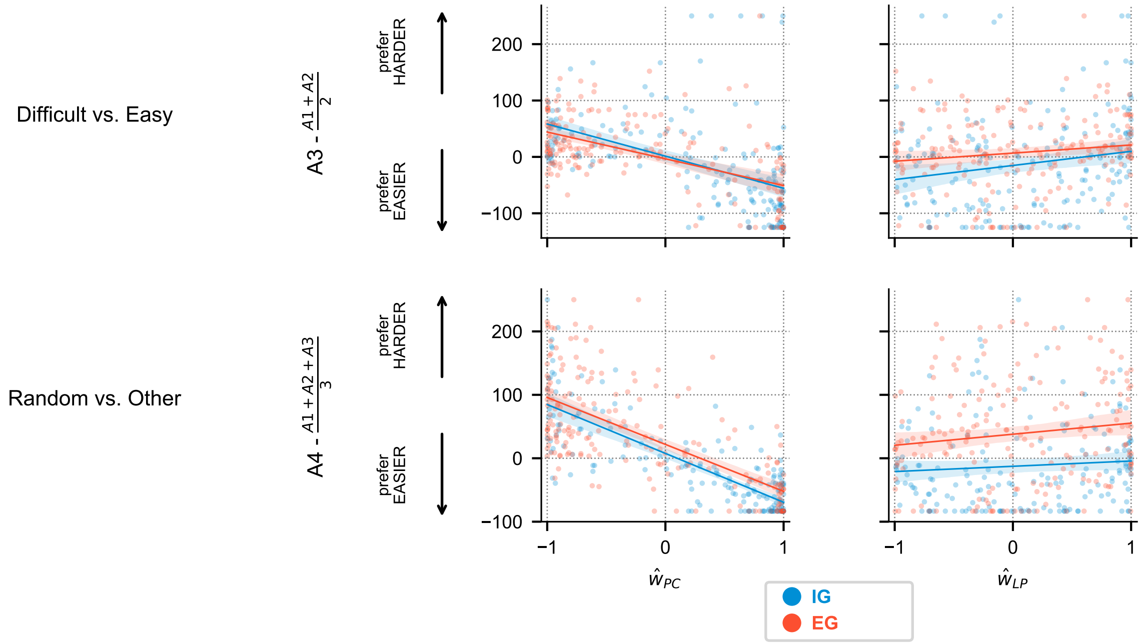

Supplementary Figure 7: Each point is one participant in the IG (blue, $N = 177$) and EG (red, $N = 188$) group. The y-axis shows the difference between the number of trials a participant chose A3 minus the average number of trials spent on A1 and A2; the bottom row compares the random activity to all other activities. The x-axis shows the normalized w_{PC} and w_{LP} coefficients from the bivariate models. The lines represent linear models of activity difficulty preference as a function of normalized coefficients (each line pair fitted separately for the corresponding subplot; shaded regions represent 95% confidence intervals). w_{PC} coefficients (negative values indicating the tolerance for errors) were associated with choices of harder activities regardless of their learnability (left column). In contrast, w_{LP} coefficients (indicating more sensitivity to performance derivatives) were positively related to a preference for a harder activity only when that activity was learnable (top right), but not when it was unlearnable (bottom right). Source data are provided as a Source Data file.

measure of familiarity is a good predictor of the choice of activity, but it does not explain the choice very well. Thus, even if familiarity was an important component for the utility-based prediction (which is indeed the case), it would not be a good explanatory variable, because it itself is determined by choices.

Here we discuss a more extensive model comparisons exercise which included the additional familiarity component on top of those reported in the main text. We operationalized familiarity as exposure (EXP) to a learning activity defined as a min-max normalized count of choice of activity. Specifically, we simply counted the number of times an activity was chosen by a participant on each trial of free play, and then re-scaled the counts to be between 0 and 1, using min-max normalization (this normalization was also applied to all PC and LP before fitting the models):

$$\text{norm}(\text{count}_{t,i}) = \frac{\text{count}_{t,i} - \min(\text{counts})}{\max(\text{counts}) - \min(\text{counts})} \qquad (6.1)$$

Where $\text{count}_{t,i}$ is the number of times on which activity i was selected prior to trial t, and $\max(counts)$ and $\min(counts)$ denote, respectively, the maximum and minimum counts across all tasks and trials.

The EXP measure was added to the set of potential utility function components for all-subsets model comparisons. Fig Supplementary Fig. 6. presents the distributions of AIC scores of each subset of variables included in the model. The full-form trivariate model (EXP + PC + LP) had the lowest AIC on average (M = 438.068, SD = 212.092). Thus, even when familiarity was included in the mix, both PC and LP were still important factors in increasing model likelihood. These results further support the importance of learning-based heuristics for the self-determined choice of activity. At the individual level, when compared to all of the other 6 model forms, the trivariate model (EXP + PC + LP) had the lowest AIC score in only 49.59% of participants (Supplementary Fig. 4) and was at least 2 points less than any other model in only 38.90% of individuals. Moreover, the median AIC scores of the trivariate model and the next best fitting model among individuals was nonsignificant ($Z(365) = 181$, $p = .917$). These results show that although the EXP component provided some further improvement in likelihood over other models, this improvement was not very substantial: the PC + LP bivariate were, on average, significantly better than univariate models, as reported in the main text.

On the other hand, models that included both PC and LP components (i.e., EXP + PC + LP and PC + LP) had the lowest AIC in 73.42% of cases. Furthermore, compared to any univariate model – including the EXP-only model – the bivariate (PC + LP) model showed reliably better AIC scores ($Z(365) = 365$, $p = .021$, Wilcoxon signed-rank test). Notwithstanding the predictive power of the EXP component alone, PC and LP components remain important predictors of self-determined activity choices.

7 Model Coefficients and Activity Preferences

As we discuss in the main text, PC and LP may play distinct roles in self-regulated learning. While PC can help learners identify challenging activities, LP can be used to avoid unlearnable activities. To examine this idea further, we analyzed how the w_{PC} and w_{LP} coefficients (normalized to reflect relative preferences as explained in the text) correlated with individual preferences for challenging over easier activities when the more challenging activity was, respectively, learnable or unlearnable. As a simple measure of the tendency to choose more challenging learnable activities, we computed the difference between the number of trials a participant devoted to activity A3 relative to the average amount of trials spent on easier activities (A1 and A2, Supplementary Fig. 7, top row). As a measure of the tendency to choose the more challenging random activity, we computed the difference between the number of trials devoted to activity A4 relative to the average amount of time spent on other activities (A1, A2, and A3; Supplementary Fig. 7, bottom row).

The w_{PC} coefficients showed negative correlations with both measures, suggesting that they captured the participants' tendency to choose more difficult tasks regardless of learnability(Supplementary Fig. 7, left). Both the preference for A3 and the preference for A4 showed negative correlations with w_{PC} (A3 vs A1&A2; IG slope = 56.969, $t(361) = -8.255$, $p < .001$; A4 vs A1-A3 slope = 86.684, $t(361) = -13.822$, $p < .001$).

In contrast, in the IG group, the w_{LP} coefficients showed a positive relationship with the preference for A3 (slope = 25.060, $t(361) = 2.817$, $p = .006$), but no relationship with the preference for A4 ($p = .356$; Supplementary Fig. 7, right), suggesting that people with higher w_{LP} coefficients tended to prefer the more difficult activity only if that activity was learnable. The EG group showed no significant relationship between w_{LP} coefficients and either measure of preference.

Given the predictions of the LP hypothesis, one might expect to actually find a negative relationship between LP and a preference for an unlearnable task, not just a lack of a relationship. Indeed, one of the appeals of the LP heuristic is that it protects the learner from fixating on low-performance activities when it is not worth it. While we did not find evidence strongly supporting or refuting this prediction, we identify two ways in which it can be obtained. First, it is possible that our rather restricted operationalization of LP was not optimal for differentiating between activities A3 and A4, which were indeed very similar in terms of their recent-feedback patterns. The LP signal was especially noisy compared to the relatively clear PC signal. Investigating a wider scope of models with alternative formulations of LP could be useful for testing the predicted preference for learnable vs unlearnable tasks. Another approach would be to implement an experimental setting similar to ours, but with a larger amount of difficult learnable and unlearnable tasks. Such a setting would be more effective in showing whether sensitivity to LP helps avoiding activities that are impossible to learn.

Supplementary References

[1] Cédric Colas, Pierre Fournier, Mohamed Chetouani, Olivier Sigaud, and Pierre-Yves Oudeyer. Curious: intrinsically motivated modular multi-goal reinforcement learning. In *International conference on machine learning*, pages 1331–1340, 2019.

[2] Nicolas Bougie and Ryutaro Ichise. Skill-based curiosity for intrinsically motivated reinforcement learning. *Machine Learning*, 109(3):493–512, 2020.

[3] Marc Bellemare, Sriram Srinivasan, Georg Ostrovski, Tom Schaul, David Saxton, and Remi Munos. Unifying count-based exploration and intrinsic motivation. In *Advances in neural information processing systems*, pages 1471–1479, 2016.
