# Supplementary material for: Humans monitor learning progress in curiosity-driven exploration
Source: Nat Commun. 2021 Oct 13;12:5972. doi: 10.1038/s41467-021-26196-w (PMC8514490; doi:10.1038/s41467-021-26196-w)
Supplement: Supplementary file 2 — Description of Additional Supplementary Files [file 41467_2021_26196_MOESM2_ESM.pdf]

Description of Additional Supplementary Files

Supplementary Software 1: Source code of the publicly released code repository.

This is a 2nd release that includes the original stimuli that were used in the experiment.
