# Supplementary material for: Humans monitor learning progress in curiosity-driven exploration
Source: Nat Commun. 2021 Oct 13;12:5972. doi: 10.1038/s41467-021-26196-w (PMC8514490; doi:10.1038/s41467-021-26196-w)
Supplement: Supplementary file 3 — Supplementary Software 1 [file 41467_2021_26196_MOESM3_ESM.zip › Humans-monitor-LP-2.0/figures/fig_s7.pdf]

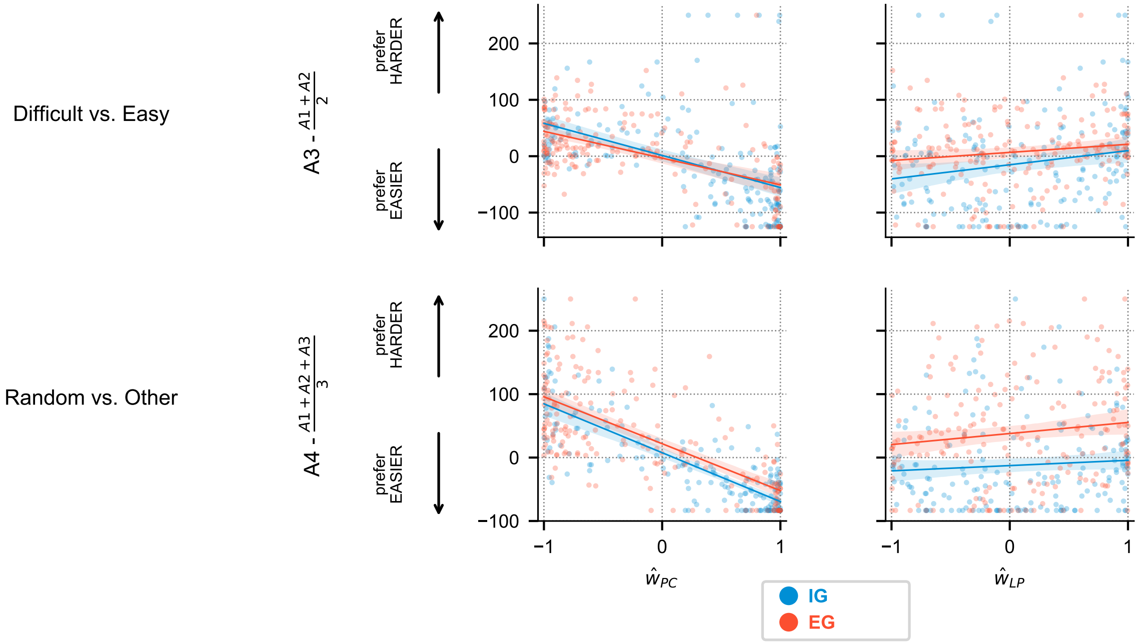

Difficult vs. Easy
Random vs. Other
A3 - $\frac{A1+A2}{2}$
A4 - $\frac{A1+A2+A3}{3}$
prefer HARDER
prefer EASIER
prefer HARDER
prefer EASIER
200
100
0
−100
−1
0
1
$\hat{w}_{PC}$
$\hat{w}_{LP}$
IG
EG
