# Supplementary figures and images for: Humans monitor learning progress in curiosity-driven exploration (part 2 of 2)
Source: Nat Commun. 2021 Oct 13;12:5972. doi: 10.1038/s41467-021-26196-w (PMC8514490; doi:10.1038/s41467-021-26196-w)

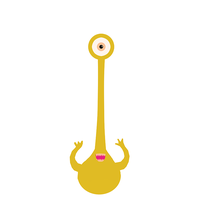

Supplement: Supplementary file 3 — Supplementary Software 1 [file 41467_2021_26196_MOESM3_ESM.zip › Humans-monitor-LP-2.0/images/s3_12_thumb.png]

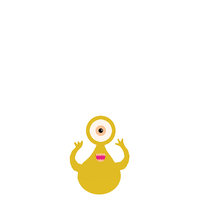

Supplement: Supplementary file 3 — Supplementary Software 1 [file 41467_2021_26196_MOESM3_ESM.zip › Humans-monitor-LP-2.0/images/s3_13_thumb.png]

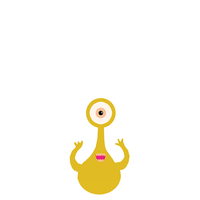

Supplement: Supplementary file 3 — Supplementary Software 1 [file 41467_2021_26196_MOESM3_ESM.zip › Humans-monitor-LP-2.0/images/s3_14_thumb.png]

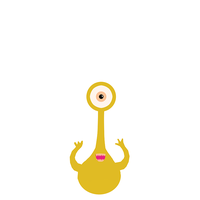

Supplement: Supplementary file 3 — Supplementary Software 1 [file 41467_2021_26196_MOESM3_ESM.zip › Humans-monitor-LP-2.0/images/s3_15_thumb.png]

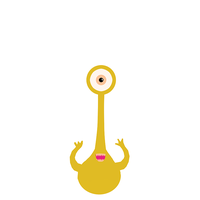

Supplement: Supplementary file 3 — Supplementary Software 1 [file 41467_2021_26196_MOESM3_ESM.zip › Humans-monitor-LP-2.0/images/s3_16_thumb.png]

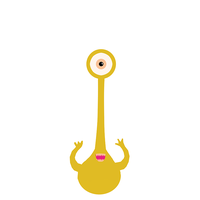

Supplement: Supplementary file 3 — Supplementary Software 1 [file 41467_2021_26196_MOESM3_ESM.zip › Humans-monitor-LP-2.0/images/s3_17_thumb.png]

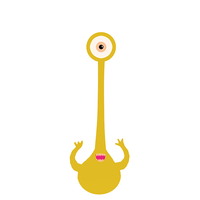

Supplement: Supplementary file 3 — Supplementary Software 1 [file 41467_2021_26196_MOESM3_ESM.zip › Humans-monitor-LP-2.0/images/s3_18_thumb.png]

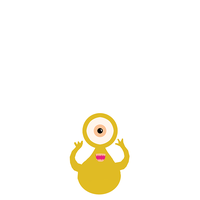

Supplement: Supplementary file 3 — Supplementary Software 1 [file 41467_2021_26196_MOESM3_ESM.zip › Humans-monitor-LP-2.0/images/s3_19_thumb.png]

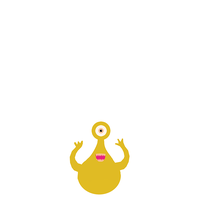

Supplement: Supplementary file 3 — Supplementary Software 1 [file 41467_2021_26196_MOESM3_ESM.zip › Humans-monitor-LP-2.0/images/s3_1_thumb.png]

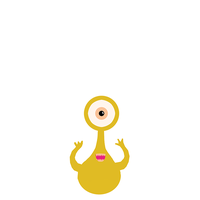

Supplement: Supplementary file 3 — Supplementary Software 1 [file 41467_2021_26196_MOESM3_ESM.zip › Humans-monitor-LP-2.0/images/s3_20_thumb.png]

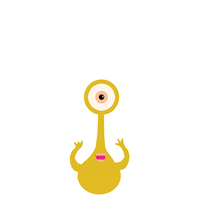

Supplement: Supplementary file 3 — Supplementary Software 1 [file 41467_2021_26196_MOESM3_ESM.zip › Humans-monitor-LP-2.0/images/s3_21_thumb.png]

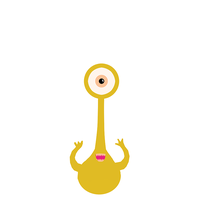

Supplement: Supplementary file 3 — Supplementary Software 1 [file 41467_2021_26196_MOESM3_ESM.zip › Humans-monitor-LP-2.0/images/s3_22_thumb.png]

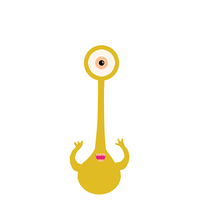

Supplement: Supplementary file 3 — Supplementary Software 1 [file 41467_2021_26196_MOESM3_ESM.zip › Humans-monitor-LP-2.0/images/s3_23_thumb.png]

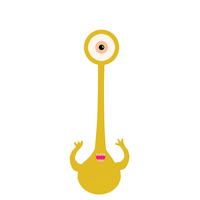

Supplement: Supplementary file 3 — Supplementary Software 1 [file 41467_2021_26196_MOESM3_ESM.zip › Humans-monitor-LP-2.0/images/s3_24_thumb.png]

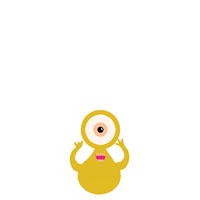

Supplement: Supplementary file 3 — Supplementary Software 1 [file 41467_2021_26196_MOESM3_ESM.zip › Humans-monitor-LP-2.0/images/s3_25_thumb.png]

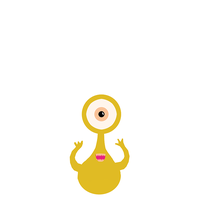

Supplement: Supplementary file 3 — Supplementary Software 1 [file 41467_2021_26196_MOESM3_ESM.zip › Humans-monitor-LP-2.0/images/s3_26_thumb.png]

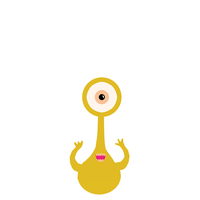

Supplement: Supplementary file 3 — Supplementary Software 1 [file 41467_2021_26196_MOESM3_ESM.zip › Humans-monitor-LP-2.0/images/s3_27_thumb.png]

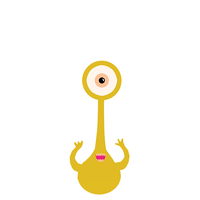

Supplement: Supplementary file 3 — Supplementary Software 1 [file 41467_2021_26196_MOESM3_ESM.zip › Humans-monitor-LP-2.0/images/s3_28_thumb.png]

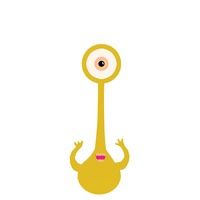

Supplement: Supplementary file 3 — Supplementary Software 1 [file 41467_2021_26196_MOESM3_ESM.zip › Humans-monitor-LP-2.0/images/s3_29_thumb.png]

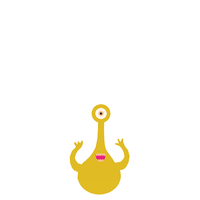

Supplement: Supplementary file 3 — Supplementary Software 1 [file 41467_2021_26196_MOESM3_ESM.zip › Humans-monitor-LP-2.0/images/s3_2_thumb.png]

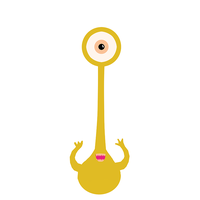

Supplement: Supplementary file 3 — Supplementary Software 1 [file 41467_2021_26196_MOESM3_ESM.zip › Humans-monitor-LP-2.0/images/s3_30_thumb.png]

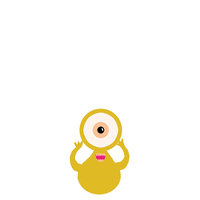

Supplement: Supplementary file 3 — Supplementary Software 1 [file 41467_2021_26196_MOESM3_ESM.zip › Humans-monitor-LP-2.0/images/s3_31_thumb.png]

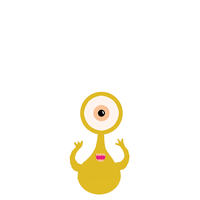

Supplement: Supplementary file 3 — Supplementary Software 1 [file 41467_2021_26196_MOESM3_ESM.zip › Humans-monitor-LP-2.0/images/s3_32_thumb.png]

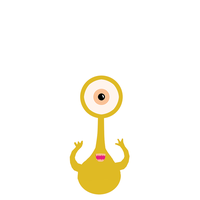

Supplement: Supplementary file 3 — Supplementary Software 1 [file 41467_2021_26196_MOESM3_ESM.zip › Humans-monitor-LP-2.0/images/s3_33_thumb.png]

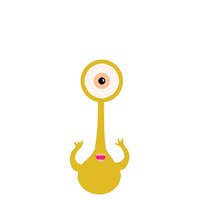

Supplement: Supplementary file 3 — Supplementary Software 1 [file 41467_2021_26196_MOESM3_ESM.zip › Humans-monitor-LP-2.0/images/s3_34_thumb.png]

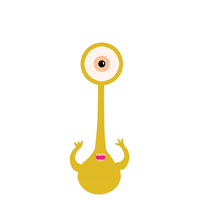

Supplement: Supplementary file 3 — Supplementary Software 1 [file 41467_2021_26196_MOESM3_ESM.zip › Humans-monitor-LP-2.0/images/s3_35_thumb.png]

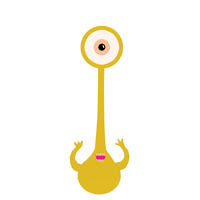

Supplement: Supplementary file 3 — Supplementary Software 1 [file 41467_2021_26196_MOESM3_ESM.zip › Humans-monitor-LP-2.0/images/s3_36_thumb.png]

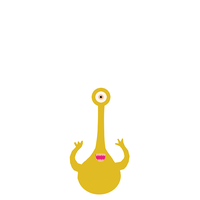

Supplement: Supplementary file 3 — Supplementary Software 1 [file 41467_2021_26196_MOESM3_ESM.zip › Humans-monitor-LP-2.0/images/s3_3_thumb.png]

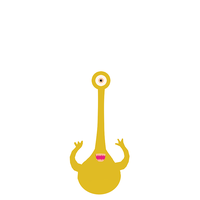

Supplement: Supplementary file 3 — Supplementary Software 1 [file 41467_2021_26196_MOESM3_ESM.zip › Humans-monitor-LP-2.0/images/s3_4_thumb.png]

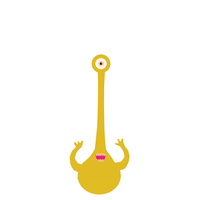

Supplement: Supplementary file 3 — Supplementary Software 1 [file 41467_2021_26196_MOESM3_ESM.zip › Humans-monitor-LP-2.0/images/s3_5_thumb.png]

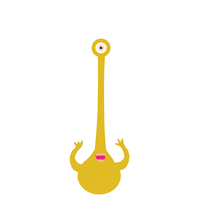

Supplement: Supplementary file 3 — Supplementary Software 1 [file 41467_2021_26196_MOESM3_ESM.zip › Humans-monitor-LP-2.0/images/s3_6_thumb.png]

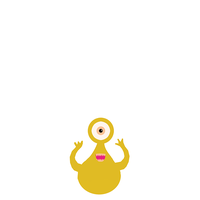

Supplement: Supplementary file 3 — Supplementary Software 1 [file 41467_2021_26196_MOESM3_ESM.zip › Humans-monitor-LP-2.0/images/s3_7_thumb.png]

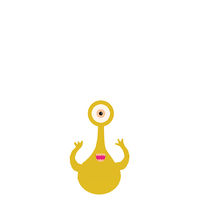

Supplement: Supplementary file 3 — Supplementary Software 1 [file 41467_2021_26196_MOESM3_ESM.zip › Humans-monitor-LP-2.0/images/s3_8_thumb.png]

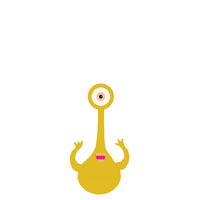

Supplement: Supplementary file 3 — Supplementary Software 1 [file 41467_2021_26196_MOESM3_ESM.zip › Humans-monitor-LP-2.0/images/s3_9_thumb.png]

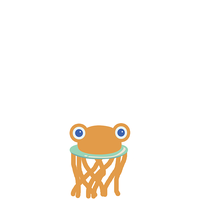

Supplement: Supplementary file 3 — Supplementary Software 1 [file 41467_2021_26196_MOESM3_ESM.zip › Humans-monitor-LP-2.0/images/s4_10_thumb.png]

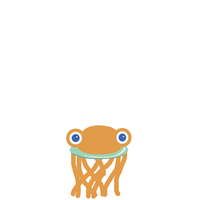

Supplement: Supplementary file 3 — Supplementary Software 1 [file 41467_2021_26196_MOESM3_ESM.zip › Humans-monitor-LP-2.0/images/s4_11_thumb.png]

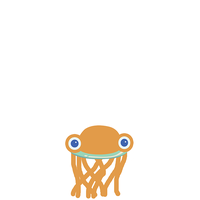

Supplement: Supplementary file 3 — Supplementary Software 1 [file 41467_2021_26196_MOESM3_ESM.zip › Humans-monitor-LP-2.0/images/s4_12_thumb.png]

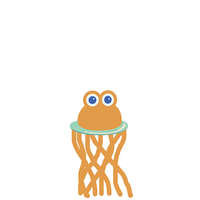

Supplement: Supplementary file 3 — Supplementary Software 1 [file 41467_2021_26196_MOESM3_ESM.zip › Humans-monitor-LP-2.0/images/s4_13_thumb.png]

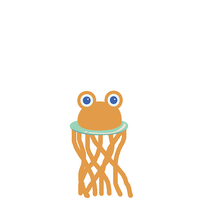

Supplement: Supplementary file 3 — Supplementary Software 1 [file 41467_2021_26196_MOESM3_ESM.zip › Humans-monitor-LP-2.0/images/s4_14_thumb.png]

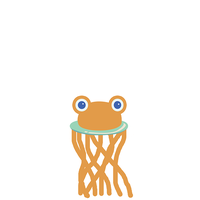

Supplement: Supplementary file 3 — Supplementary Software 1 [file 41467_2021_26196_MOESM3_ESM.zip › Humans-monitor-LP-2.0/images/s4_15_thumb.png]

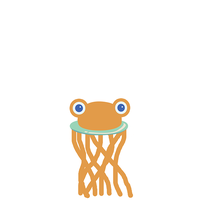

Supplement: Supplementary file 3 — Supplementary Software 1 [file 41467_2021_26196_MOESM3_ESM.zip › Humans-monitor-LP-2.0/images/s4_16_thumb.png]

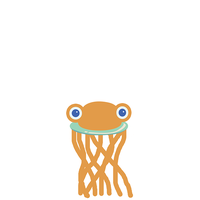

Supplement: Supplementary file 3 — Supplementary Software 1 [file 41467_2021_26196_MOESM3_ESM.zip › Humans-monitor-LP-2.0/images/s4_17_thumb.png]

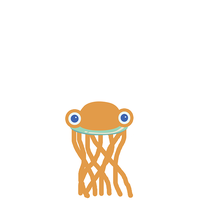

Supplement: Supplementary file 3 — Supplementary Software 1 [file 41467_2021_26196_MOESM3_ESM.zip › Humans-monitor-LP-2.0/images/s4_18_thumb.png]

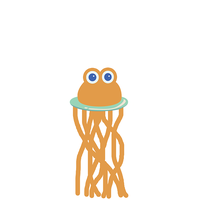

Supplement: Supplementary file 3 — Supplementary Software 1 [file 41467_2021_26196_MOESM3_ESM.zip › Humans-monitor-LP-2.0/images/s4_19_thumb.png]

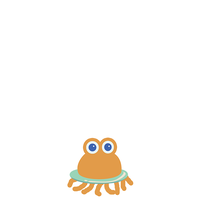

Supplement: Supplementary file 3 — Supplementary Software 1 [file 41467_2021_26196_MOESM3_ESM.zip › Humans-monitor-LP-2.0/images/s4_1_thumb.png]

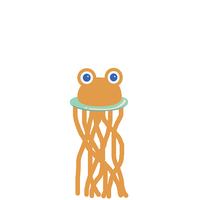

Supplement: Supplementary file 3 — Supplementary Software 1 [file 41467_2021_26196_MOESM3_ESM.zip › Humans-monitor-LP-2.0/images/s4_20_thumb.png]

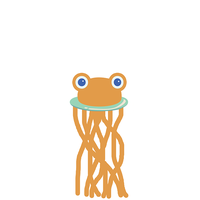

Supplement: Supplementary file 3 — Supplementary Software 1 [file 41467_2021_26196_MOESM3_ESM.zip › Humans-monitor-LP-2.0/images/s4_21_thumb.png]

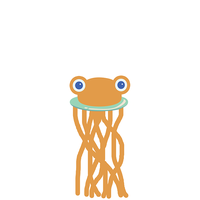

Supplement: Supplementary file 3 — Supplementary Software 1 [file 41467_2021_26196_MOESM3_ESM.zip › Humans-monitor-LP-2.0/images/s4_22_thumb.png]

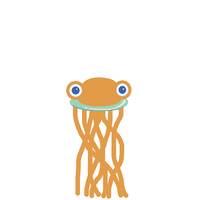

Supplement: Supplementary file 3 — Supplementary Software 1 [file 41467_2021_26196_MOESM3_ESM.zip › Humans-monitor-LP-2.0/images/s4_23_thumb.png]

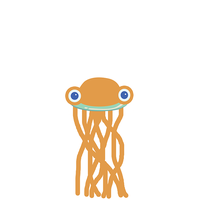

Supplement: Supplementary file 3 — Supplementary Software 1 [file 41467_2021_26196_MOESM3_ESM.zip › Humans-monitor-LP-2.0/images/s4_24_thumb.png]

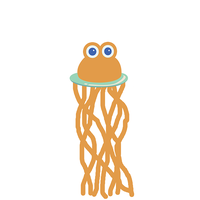

Supplement: Supplementary file 3 — Supplementary Software 1 [file 41467_2021_26196_MOESM3_ESM.zip › Humans-monitor-LP-2.0/images/s4_25_thumb.png]

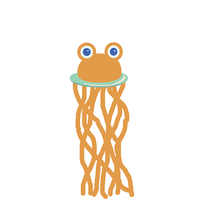

Supplement: Supplementary file 3 — Supplementary Software 1 [file 41467_2021_26196_MOESM3_ESM.zip › Humans-monitor-LP-2.0/images/s4_26_thumb.png]

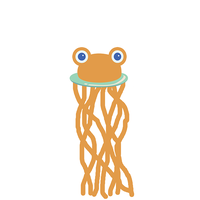

Supplement: Supplementary file 3 — Supplementary Software 1 [file 41467_2021_26196_MOESM3_ESM.zip › Humans-monitor-LP-2.0/images/s4_27_thumb.png]

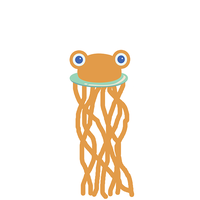

Supplement: Supplementary file 3 — Supplementary Software 1 [file 41467_2021_26196_MOESM3_ESM.zip › Humans-monitor-LP-2.0/images/s4_28_thumb.png]

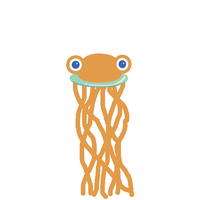

Supplement: Supplementary file 3 — Supplementary Software 1 [file 41467_2021_26196_MOESM3_ESM.zip › Humans-monitor-LP-2.0/images/s4_29_thumb.png]

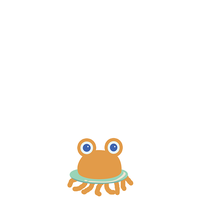

Supplement: Supplementary file 3 — Supplementary Software 1 [file 41467_2021_26196_MOESM3_ESM.zip › Humans-monitor-LP-2.0/images/s4_2_thumb.png]

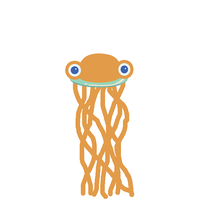

Supplement: Supplementary file 3 — Supplementary Software 1 [file 41467_2021_26196_MOESM3_ESM.zip › Humans-monitor-LP-2.0/images/s4_30_thumb.png]

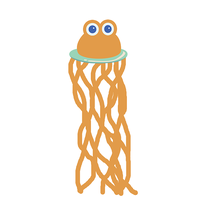

Supplement: Supplementary file 3 — Supplementary Software 1 [file 41467_2021_26196_MOESM3_ESM.zip › Humans-monitor-LP-2.0/images/s4_31_thumb.png]

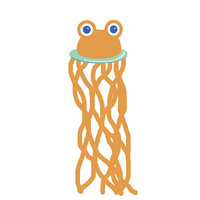

Supplement: Supplementary file 3 — Supplementary Software 1 [file 41467_2021_26196_MOESM3_ESM.zip › Humans-monitor-LP-2.0/images/s4_32_thumb.png]

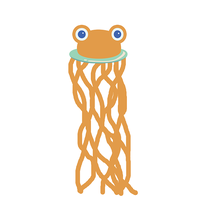

Supplement: Supplementary file 3 — Supplementary Software 1 [file 41467_2021_26196_MOESM3_ESM.zip › Humans-monitor-LP-2.0/images/s4_33_thumb.png]

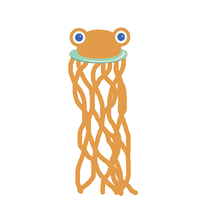

Supplement: Supplementary file 3 — Supplementary Software 1 [file 41467_2021_26196_MOESM3_ESM.zip › Humans-monitor-LP-2.0/images/s4_34_thumb.png]

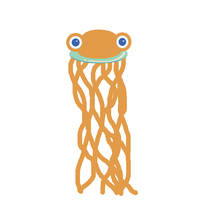

Supplement: Supplementary file 3 — Supplementary Software 1 [file 41467_2021_26196_MOESM3_ESM.zip › Humans-monitor-LP-2.0/images/s4_35_thumb.png]

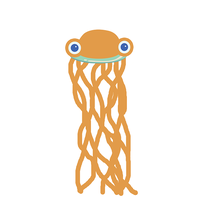

Supplement: Supplementary file 3 — Supplementary Software 1 [file 41467_2021_26196_MOESM3_ESM.zip › Humans-monitor-LP-2.0/images/s4_36_thumb.png]

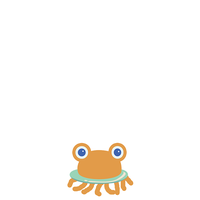

Supplement: Supplementary file 3 — Supplementary Software 1 [file 41467_2021_26196_MOESM3_ESM.zip › Humans-monitor-LP-2.0/images/s4_3_thumb.png]

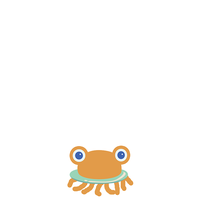

Supplement: Supplementary file 3 — Supplementary Software 1 [file 41467_2021_26196_MOESM3_ESM.zip › Humans-monitor-LP-2.0/images/s4_4_thumb.png]

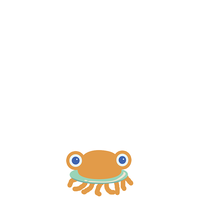

Supplement: Supplementary file 3 — Supplementary Software 1 [file 41467_2021_26196_MOESM3_ESM.zip › Humans-monitor-LP-2.0/images/s4_5_thumb.png]

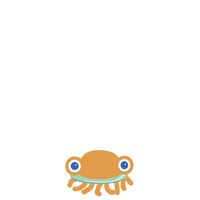

Supplement: Supplementary file 3 — Supplementary Software 1 [file 41467_2021_26196_MOESM3_ESM.zip › Humans-monitor-LP-2.0/images/s4_6_thumb.png]

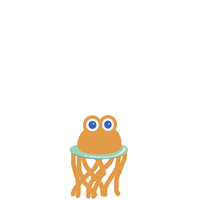

Supplement: Supplementary file 3 — Supplementary Software 1 [file 41467_2021_26196_MOESM3_ESM.zip › Humans-monitor-LP-2.0/images/s4_7_thumb.png]

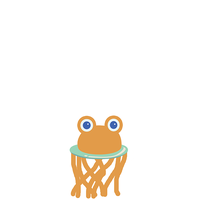

Supplement: Supplementary file 3 — Supplementary Software 1 [file 41467_2021_26196_MOESM3_ESM.zip › Humans-monitor-LP-2.0/images/s4_8_thumb.png]

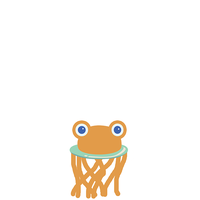

Supplement: Supplementary file 3 — Supplementary Software 1 [file 41467_2021_26196_MOESM3_ESM.zip › Humans-monitor-LP-2.0/images/s4_9_thumb.png]

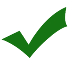

Supplement: Supplementary file 3 — Supplementary Software 1 [file 41467_2021_26196_MOESM3_ESM.zip › Humans-monitor-LP-2.0/stimuli/feedback/greencheck.png]

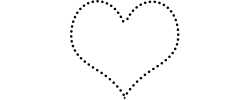

Supplement: Supplementary file 3 — Supplementary Software 1 [file 41467_2021_26196_MOESM3_ESM.zip › Humans-monitor-LP-2.0/stimuli/feedback/heart-thought.png]

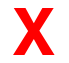

Supplement: Supplementary file 3 — Supplementary Software 1 [file 41467_2021_26196_MOESM3_ESM.zip › Humans-monitor-LP-2.0/stimuli/feedback/redx.png]

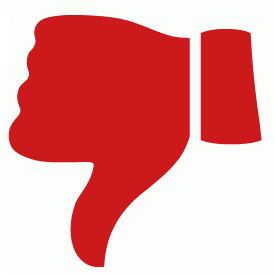

Supplement: Supplementary file 3 — Supplementary Software 1 [file 41467_2021_26196_MOESM3_ESM.zip › Humans-monitor-LP-2.0/stimuli/feedback/thumbs-down.png]

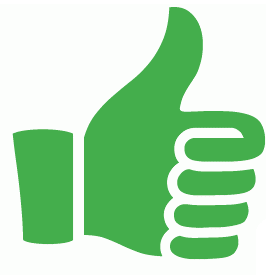

Supplement: Supplementary file 3 — Supplementary Software 1 [file 41467_2021_26196_MOESM3_ESM.zip › Humans-monitor-LP-2.0/stimuli/feedback/thumbs-up.png]

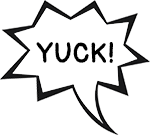

Supplement: Supplementary file 3 — Supplementary Software 1 [file 41467_2021_26196_MOESM3_ESM.zip › Humans-monitor-LP-2.0/stimuli/feedback/yuck.png]

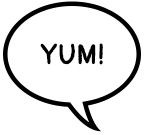

Supplement: Supplementary file 3 — Supplementary Software 1 [file 41467_2021_26196_MOESM3_ESM.zip › Humans-monitor-LP-2.0/stimuli/feedback/yum.png]

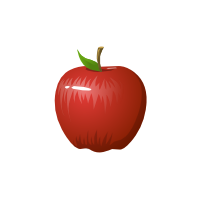

Supplement: Supplementary file 3 — Supplementary Software 1 [file 41467_2021_26196_MOESM3_ESM.zip › Humans-monitor-LP-2.0/stimuli/food/apple.png]

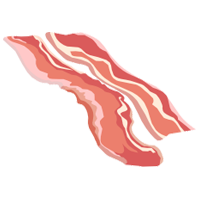

Supplement: Supplementary file 3 — Supplementary Software 1 [file 41467_2021_26196_MOESM3_ESM.zip › Humans-monitor-LP-2.0/stimuli/food/bacon.png]

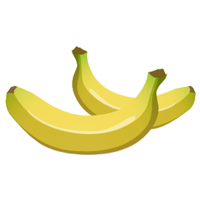

Supplement: Supplementary file 3 — Supplementary Software 1 [file 41467_2021_26196_MOESM3_ESM.zip › Humans-monitor-LP-2.0/stimuli/food/bananas.png]

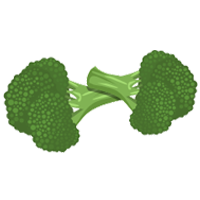

Supplement: Supplementary file 3 — Supplementary Software 1 [file 41467_2021_26196_MOESM3_ESM.zip › Humans-monitor-LP-2.0/stimuli/food/broccoli.png]

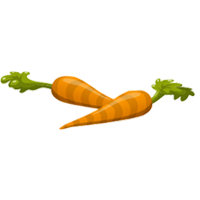

Supplement: Supplementary file 3 — Supplementary Software 1 [file 41467_2021_26196_MOESM3_ESM.zip › Humans-monitor-LP-2.0/stimuli/food/carrot.png]

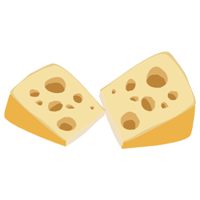

Supplement: Supplementary file 3 — Supplementary Software 1 [file 41467_2021_26196_MOESM3_ESM.zip › Humans-monitor-LP-2.0/stimuli/food/cheese.png]

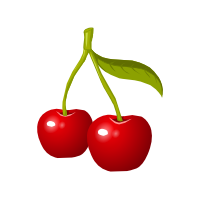

Supplement: Supplementary file 3 — Supplementary Software 1 [file 41467_2021_26196_MOESM3_ESM.zip › Humans-monitor-LP-2.0/stimuli/food/cherry.png]

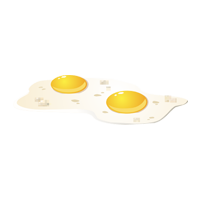

Supplement: Supplementary file 3 — Supplementary Software 1 [file 41467_2021_26196_MOESM3_ESM.zip › Humans-monitor-LP-2.0/stimuli/food/fried_eggs.png]
