# Supplementary figures and images for: Humans monitor learning progress in curiosity-driven exploration (part 1 of 2)
Source: Nat Commun. 2021 Oct 13;12:5972. doi: 10.1038/s41467-021-26196-w (PMC8514490; doi:10.1038/s41467-021-26196-w)

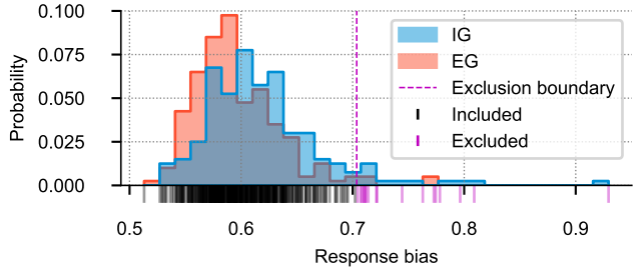

Supplement: Supplementary file 3 — Supplementary Software 1 [file 41467_2021_26196_MOESM3_ESM.zip › Humans-monitor-LP-2.0/figures/fig_s1.pdf]

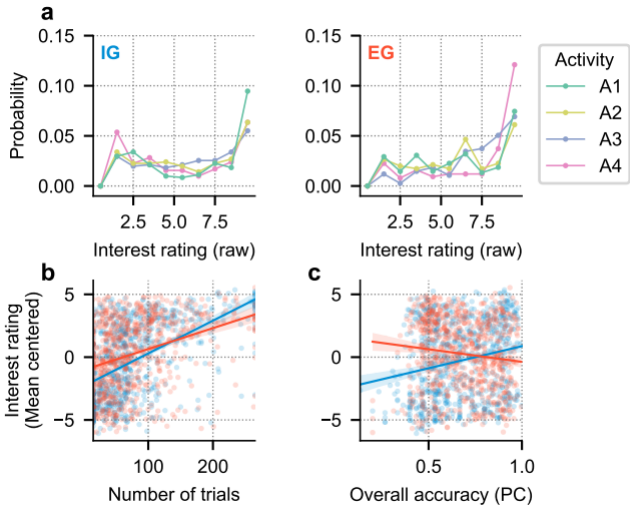

Supplement: Supplementary file 3 — Supplementary Software 1 [file 41467_2021_26196_MOESM3_ESM.zip › Humans-monitor-LP-2.0/figures/fig_s2.pdf]

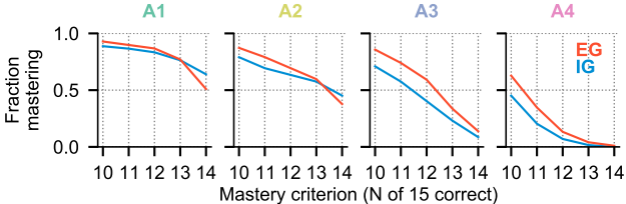

Supplement: Supplementary file 3 — Supplementary Software 1 [file 41467_2021_26196_MOESM3_ESM.zip › Humans-monitor-LP-2.0/figures/fig_s3.pdf]

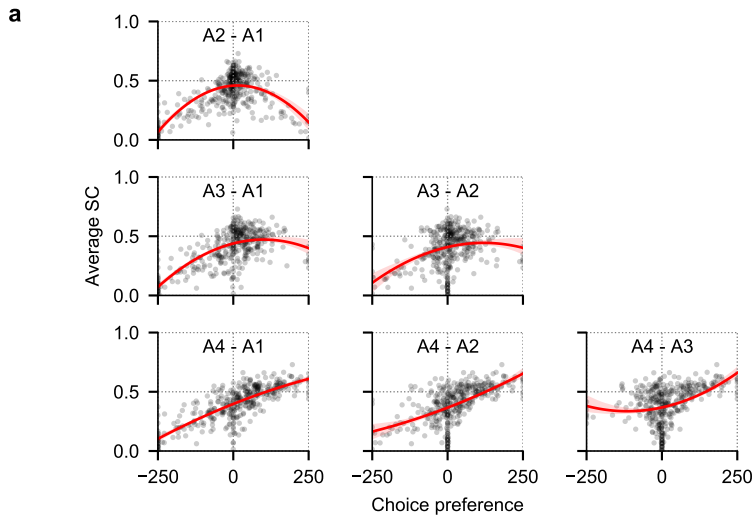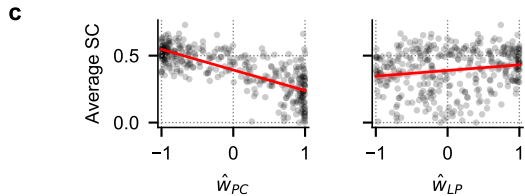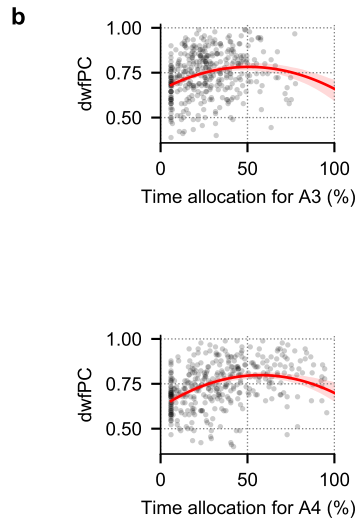

Supplement: Supplementary file 3 — Supplementary Software 1 [file 41467_2021_26196_MOESM3_ESM.zip › Humans-monitor-LP-2.0/figures/fig_s4.pdf]

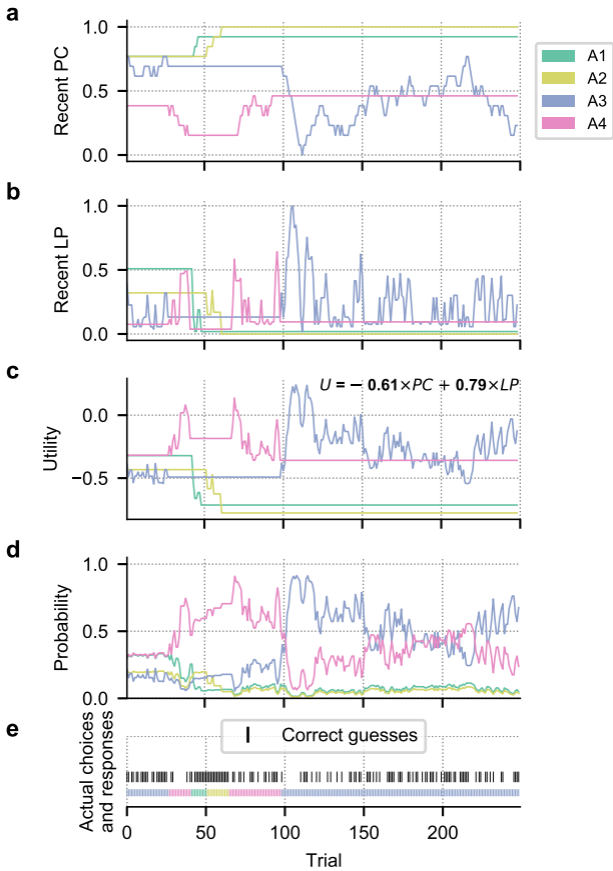

Supplement: Supplementary file 3 — Supplementary Software 1 [file 41467_2021_26196_MOESM3_ESM.zip › Humans-monitor-LP-2.0/figures/fig_s5.pdf]

Model form

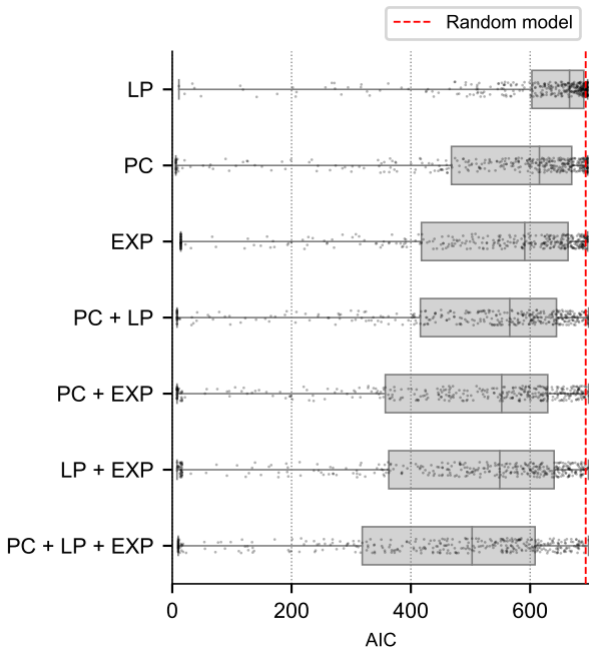

Supplement: Supplementary file 3 — Supplementary Software 1 [file 41467_2021_26196_MOESM3_ESM.zip › Humans-monitor-LP-2.0/figures/fig_s6.pdf]

**a**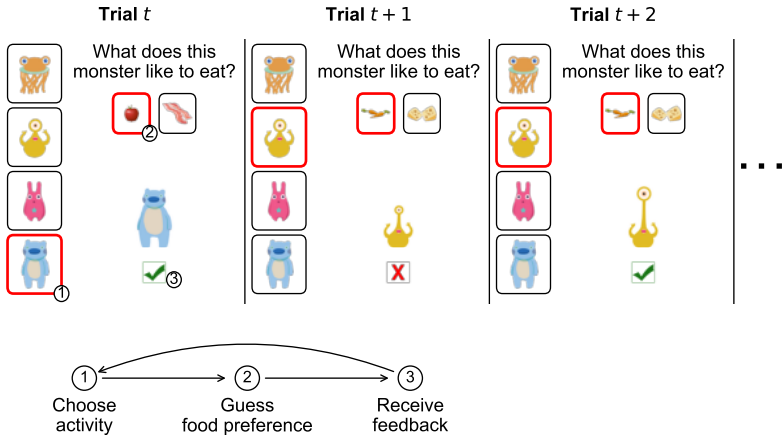**b**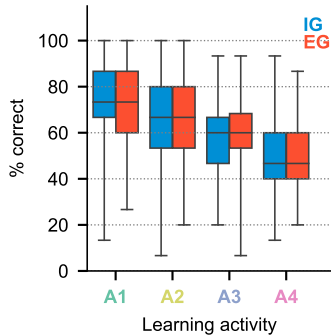

Supplement: Supplementary file 3 — Supplementary Software 1 [file 41467_2021_26196_MOESM3_ESM.zip › Humans-monitor-LP-2.0/figures/figure1.pdf]

**a**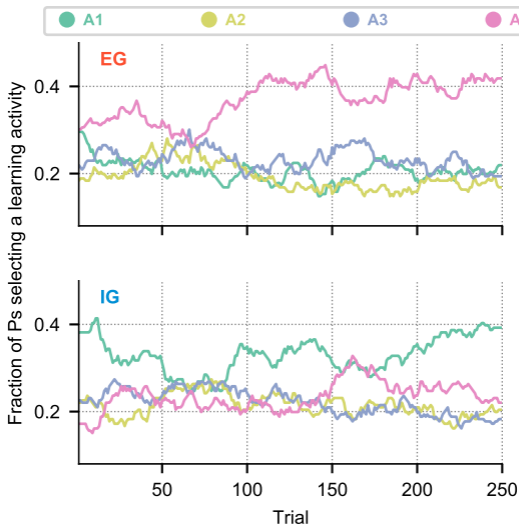**b**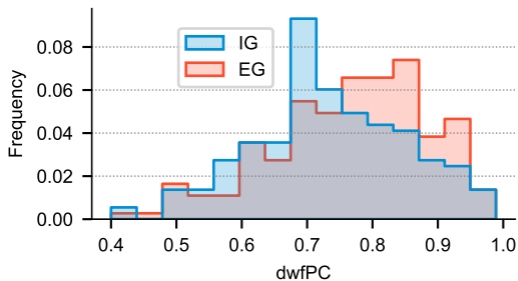

Supplement: Supplementary file 3 — Supplementary Software 1 [file 41467_2021_26196_MOESM3_ESM.zip › Humans-monitor-LP-2.0/figures/figure2.pdf]

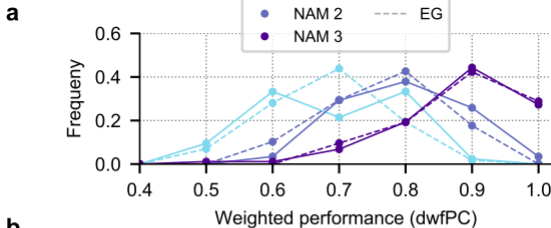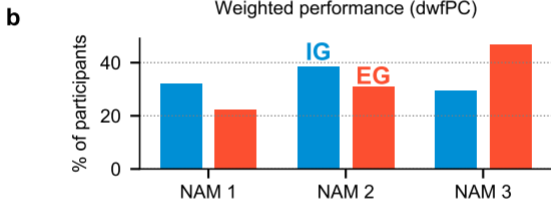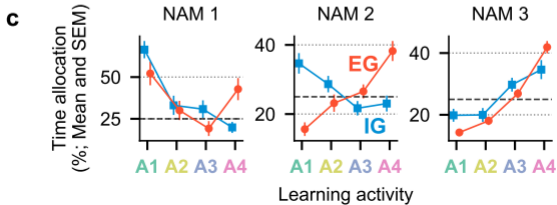

Supplement: Supplementary file 3 — Supplementary Software 1 [file 41467_2021_26196_MOESM3_ESM.zip › Humans-monitor-LP-2.0/figures/figure3.pdf]

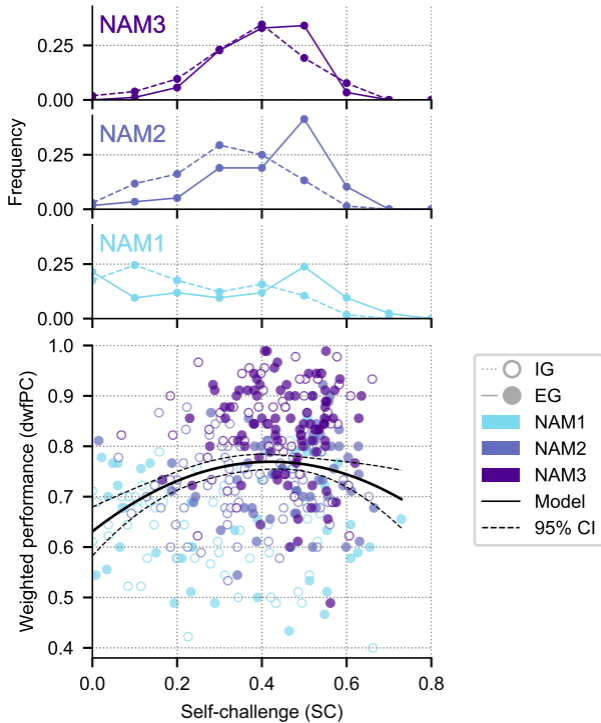

Supplement: Supplementary file 3 — Supplementary Software 1 [file 41467_2021_26196_MOESM3_ESM.zip › Humans-monitor-LP-2.0/figures/figure4.pdf]

**a**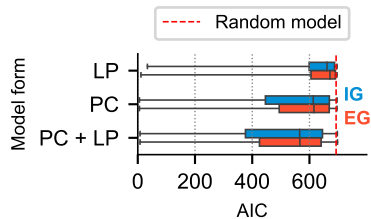**b**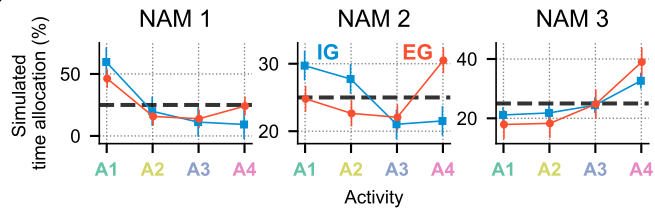**c**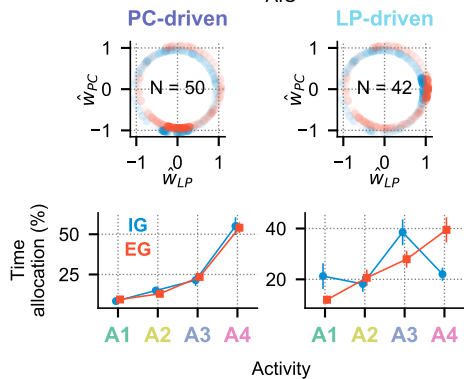**d**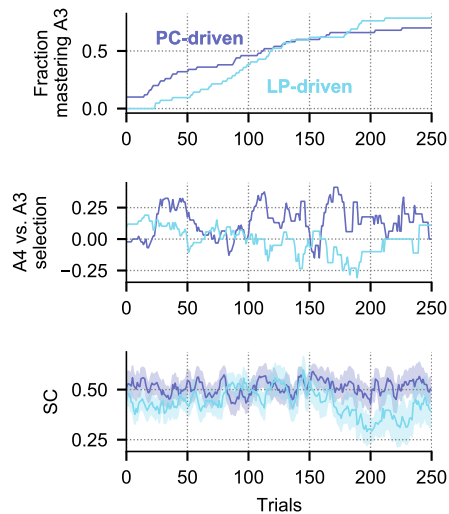

Supplement: Supplementary file 3 — Supplementary Software 1 [file 41467_2021_26196_MOESM3_ESM.zip › Humans-monitor-LP-2.0/figures/figure5.pdf]

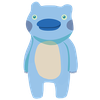

Supplement: Supplementary file 3 — Supplementary Software 1 [file 41467_2021_26196_MOESM3_ESM.zip › Humans-monitor-LP-2.0/images/c1_thumb.png]

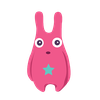

Supplement: Supplementary file 3 — Supplementary Software 1 [file 41467_2021_26196_MOESM3_ESM.zip › Humans-monitor-LP-2.0/images/c2_thumb.png]

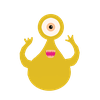

Supplement: Supplementary file 3 — Supplementary Software 1 [file 41467_2021_26196_MOESM3_ESM.zip › Humans-monitor-LP-2.0/images/c3_thumb.png]

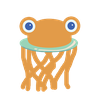

Supplement: Supplementary file 3 — Supplementary Software 1 [file 41467_2021_26196_MOESM3_ESM.zip › Humans-monitor-LP-2.0/images/c4_thumb.png]

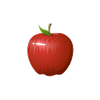

Supplement: Supplementary file 3 — Supplementary Software 1 [file 41467_2021_26196_MOESM3_ESM.zip › Humans-monitor-LP-2.0/images/f11_thumb.png]

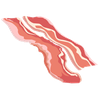

Supplement: Supplementary file 3 — Supplementary Software 1 [file 41467_2021_26196_MOESM3_ESM.zip › Humans-monitor-LP-2.0/images/f12_thumb.png]

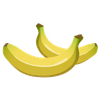

Supplement: Supplementary file 3 — Supplementary Software 1 [file 41467_2021_26196_MOESM3_ESM.zip › Humans-monitor-LP-2.0/images/f21_thumb.png]

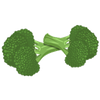

Supplement: Supplementary file 3 — Supplementary Software 1 [file 41467_2021_26196_MOESM3_ESM.zip › Humans-monitor-LP-2.0/images/f22_thumb.png]

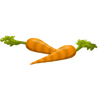

Supplement: Supplementary file 3 — Supplementary Software 1 [file 41467_2021_26196_MOESM3_ESM.zip › Humans-monitor-LP-2.0/images/f31_thumb.png]

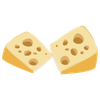

Supplement: Supplementary file 3 — Supplementary Software 1 [file 41467_2021_26196_MOESM3_ESM.zip › Humans-monitor-LP-2.0/images/f32_thumb.png]

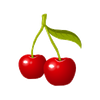

Supplement: Supplementary file 3 — Supplementary Software 1 [file 41467_2021_26196_MOESM3_ESM.zip › Humans-monitor-LP-2.0/images/f41_thumb.png]

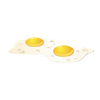

Supplement: Supplementary file 3 — Supplementary Software 1 [file 41467_2021_26196_MOESM3_ESM.zip › Humans-monitor-LP-2.0/images/f42_thumb.png]

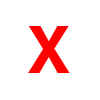

Supplement: Supplementary file 3 — Supplementary Software 1 [file 41467_2021_26196_MOESM3_ESM.zip › Humans-monitor-LP-2.0/images/fneg_thumb.png]

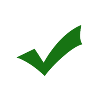

Supplement: Supplementary file 3 — Supplementary Software 1 [file 41467_2021_26196_MOESM3_ESM.zip › Humans-monitor-LP-2.0/images/fpos_thumb.png]

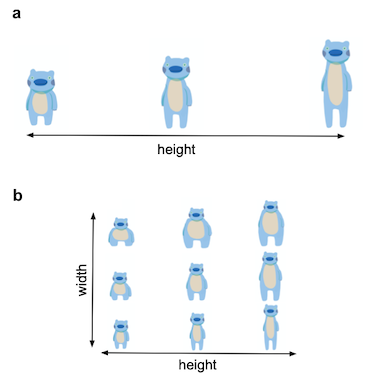

Supplement: Supplementary file 3 — Supplementary Software 1 [file 41467_2021_26196_MOESM3_ESM.zip › Humans-monitor-LP-2.0/images/monster_dimensions.png]

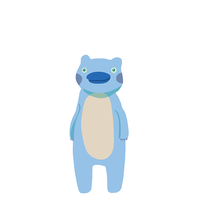

Supplement: Supplementary file 3 — Supplementary Software 1 [file 41467_2021_26196_MOESM3_ESM.zip › Humans-monitor-LP-2.0/images/s1_10_thumb.png]

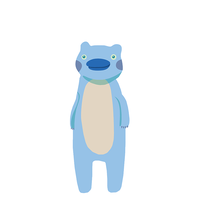

Supplement: Supplementary file 3 — Supplementary Software 1 [file 41467_2021_26196_MOESM3_ESM.zip › Humans-monitor-LP-2.0/images/s1_11_thumb.png]

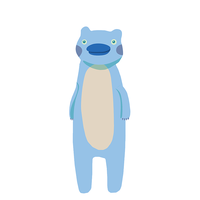

Supplement: Supplementary file 3 — Supplementary Software 1 [file 41467_2021_26196_MOESM3_ESM.zip › Humans-monitor-LP-2.0/images/s1_12_thumb.png]

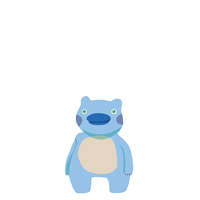

Supplement: Supplementary file 3 — Supplementary Software 1 [file 41467_2021_26196_MOESM3_ESM.zip › Humans-monitor-LP-2.0/images/s1_13_thumb.png]

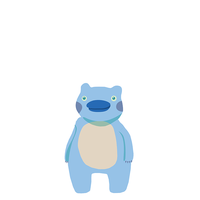

Supplement: Supplementary file 3 — Supplementary Software 1 [file 41467_2021_26196_MOESM3_ESM.zip › Humans-monitor-LP-2.0/images/s1_14_thumb.png]

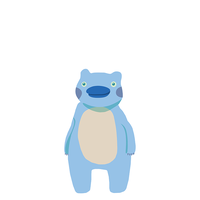

Supplement: Supplementary file 3 — Supplementary Software 1 [file 41467_2021_26196_MOESM3_ESM.zip › Humans-monitor-LP-2.0/images/s1_15_thumb.png]

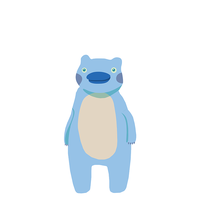

Supplement: Supplementary file 3 — Supplementary Software 1 [file 41467_2021_26196_MOESM3_ESM.zip › Humans-monitor-LP-2.0/images/s1_16_thumb.png]

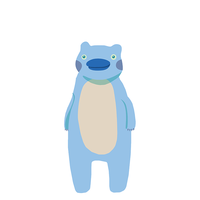

Supplement: Supplementary file 3 — Supplementary Software 1 [file 41467_2021_26196_MOESM3_ESM.zip › Humans-monitor-LP-2.0/images/s1_17_thumb.png]

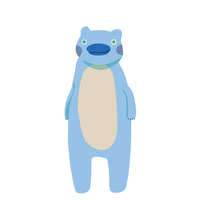

Supplement: Supplementary file 3 — Supplementary Software 1 [file 41467_2021_26196_MOESM3_ESM.zip › Humans-monitor-LP-2.0/images/s1_18_thumb.png]

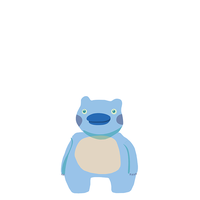

Supplement: Supplementary file 3 — Supplementary Software 1 [file 41467_2021_26196_MOESM3_ESM.zip › Humans-monitor-LP-2.0/images/s1_19_thumb.png]

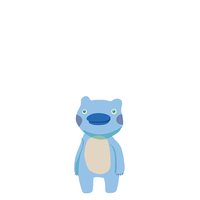

Supplement: Supplementary file 3 — Supplementary Software 1 [file 41467_2021_26196_MOESM3_ESM.zip › Humans-monitor-LP-2.0/images/s1_1_thumb.png]

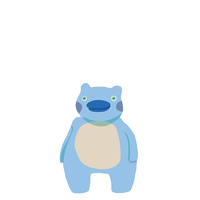

Supplement: Supplementary file 3 — Supplementary Software 1 [file 41467_2021_26196_MOESM3_ESM.zip › Humans-monitor-LP-2.0/images/s1_20_thumb.png]

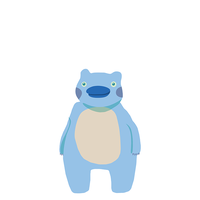

Supplement: Supplementary file 3 — Supplementary Software 1 [file 41467_2021_26196_MOESM3_ESM.zip › Humans-monitor-LP-2.0/images/s1_21_thumb.png]

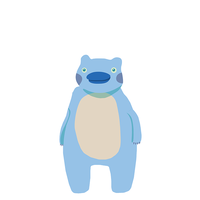

Supplement: Supplementary file 3 — Supplementary Software 1 [file 41467_2021_26196_MOESM3_ESM.zip › Humans-monitor-LP-2.0/images/s1_22_thumb.png]

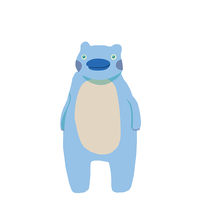

Supplement: Supplementary file 3 — Supplementary Software 1 [file 41467_2021_26196_MOESM3_ESM.zip › Humans-monitor-LP-2.0/images/s1_23_thumb.png]

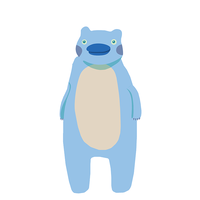

Supplement: Supplementary file 3 — Supplementary Software 1 [file 41467_2021_26196_MOESM3_ESM.zip › Humans-monitor-LP-2.0/images/s1_24_thumb.png]

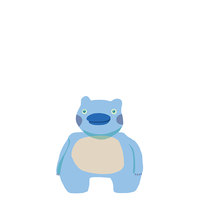

Supplement: Supplementary file 3 — Supplementary Software 1 [file 41467_2021_26196_MOESM3_ESM.zip › Humans-monitor-LP-2.0/images/s1_25_thumb.png]

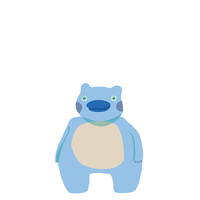

Supplement: Supplementary file 3 — Supplementary Software 1 [file 41467_2021_26196_MOESM3_ESM.zip › Humans-monitor-LP-2.0/images/s1_26_thumb.png]

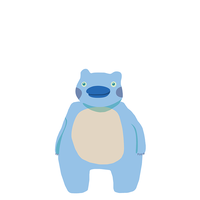

Supplement: Supplementary file 3 — Supplementary Software 1 [file 41467_2021_26196_MOESM3_ESM.zip › Humans-monitor-LP-2.0/images/s1_27_thumb.png]

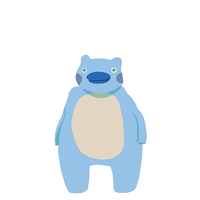

Supplement: Supplementary file 3 — Supplementary Software 1 [file 41467_2021_26196_MOESM3_ESM.zip › Humans-monitor-LP-2.0/images/s1_28_thumb.png]

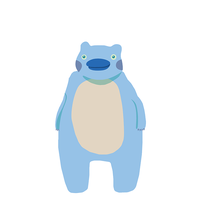

Supplement: Supplementary file 3 — Supplementary Software 1 [file 41467_2021_26196_MOESM3_ESM.zip › Humans-monitor-LP-2.0/images/s1_29_thumb.png]

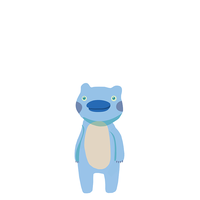

Supplement: Supplementary file 3 — Supplementary Software 1 [file 41467_2021_26196_MOESM3_ESM.zip › Humans-monitor-LP-2.0/images/s1_2_thumb.png]

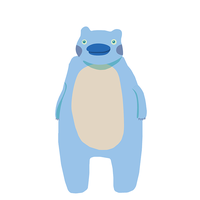

Supplement: Supplementary file 3 — Supplementary Software 1 [file 41467_2021_26196_MOESM3_ESM.zip › Humans-monitor-LP-2.0/images/s1_30_thumb.png]

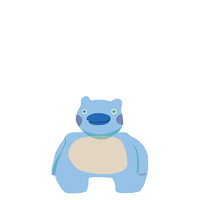

Supplement: Supplementary file 3 — Supplementary Software 1 [file 41467_2021_26196_MOESM3_ESM.zip › Humans-monitor-LP-2.0/images/s1_31_thumb.png]

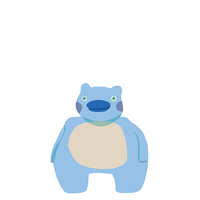

Supplement: Supplementary file 3 — Supplementary Software 1 [file 41467_2021_26196_MOESM3_ESM.zip › Humans-monitor-LP-2.0/images/s1_32_thumb.png]

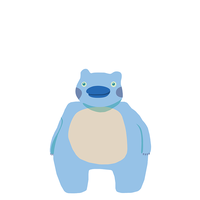

Supplement: Supplementary file 3 — Supplementary Software 1 [file 41467_2021_26196_MOESM3_ESM.zip › Humans-monitor-LP-2.0/images/s1_33_thumb.png]

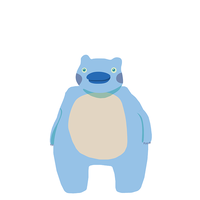

Supplement: Supplementary file 3 — Supplementary Software 1 [file 41467_2021_26196_MOESM3_ESM.zip › Humans-monitor-LP-2.0/images/s1_34_thumb.png]

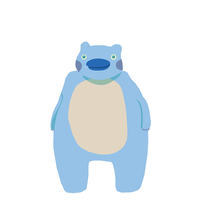

Supplement: Supplementary file 3 — Supplementary Software 1 [file 41467_2021_26196_MOESM3_ESM.zip › Humans-monitor-LP-2.0/images/s1_35_thumb.png]

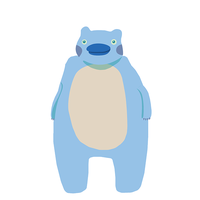

Supplement: Supplementary file 3 — Supplementary Software 1 [file 41467_2021_26196_MOESM3_ESM.zip › Humans-monitor-LP-2.0/images/s1_36_thumb.png]

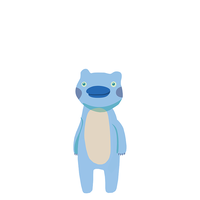

Supplement: Supplementary file 3 — Supplementary Software 1 [file 41467_2021_26196_MOESM3_ESM.zip › Humans-monitor-LP-2.0/images/s1_3_thumb.png]

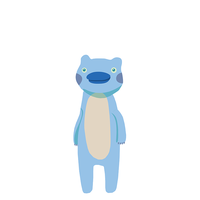

Supplement: Supplementary file 3 — Supplementary Software 1 [file 41467_2021_26196_MOESM3_ESM.zip › Humans-monitor-LP-2.0/images/s1_4_thumb.png]

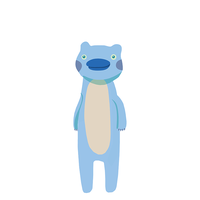

Supplement: Supplementary file 3 — Supplementary Software 1 [file 41467_2021_26196_MOESM3_ESM.zip › Humans-monitor-LP-2.0/images/s1_5_thumb.png]

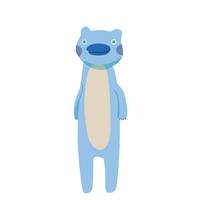

Supplement: Supplementary file 3 — Supplementary Software 1 [file 41467_2021_26196_MOESM3_ESM.zip › Humans-monitor-LP-2.0/images/s1_6_thumb.png]

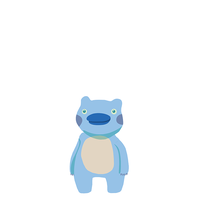

Supplement: Supplementary file 3 — Supplementary Software 1 [file 41467_2021_26196_MOESM3_ESM.zip › Humans-monitor-LP-2.0/images/s1_7_thumb.png]

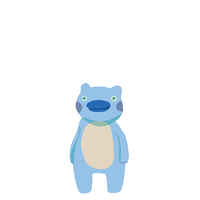

Supplement: Supplementary file 3 — Supplementary Software 1 [file 41467_2021_26196_MOESM3_ESM.zip › Humans-monitor-LP-2.0/images/s1_8_thumb.png]

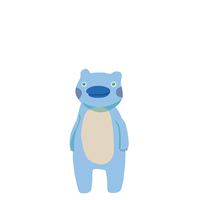

Supplement: Supplementary file 3 — Supplementary Software 1 [file 41467_2021_26196_MOESM3_ESM.zip › Humans-monitor-LP-2.0/images/s1_9_thumb.png]

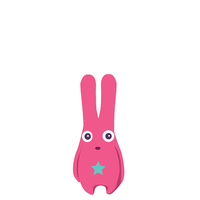

Supplement: Supplementary file 3 — Supplementary Software 1 [file 41467_2021_26196_MOESM3_ESM.zip › Humans-monitor-LP-2.0/images/s2_10_thumb.png]

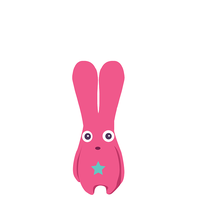

Supplement: Supplementary file 3 — Supplementary Software 1 [file 41467_2021_26196_MOESM3_ESM.zip › Humans-monitor-LP-2.0/images/s2_11_thumb.png]

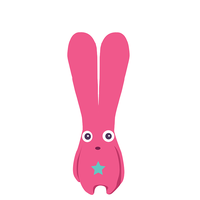

Supplement: Supplementary file 3 — Supplementary Software 1 [file 41467_2021_26196_MOESM3_ESM.zip › Humans-monitor-LP-2.0/images/s2_12_thumb.png]

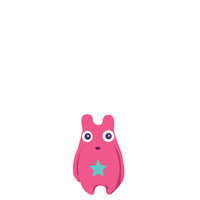

Supplement: Supplementary file 3 — Supplementary Software 1 [file 41467_2021_26196_MOESM3_ESM.zip › Humans-monitor-LP-2.0/images/s2_13_thumb.png]

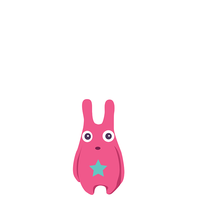

Supplement: Supplementary file 3 — Supplementary Software 1 [file 41467_2021_26196_MOESM3_ESM.zip › Humans-monitor-LP-2.0/images/s2_14_thumb.png]

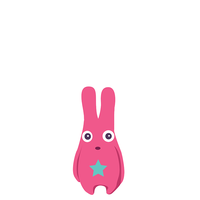

Supplement: Supplementary file 3 — Supplementary Software 1 [file 41467_2021_26196_MOESM3_ESM.zip › Humans-monitor-LP-2.0/images/s2_15_thumb.png]

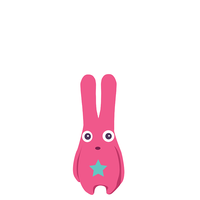

Supplement: Supplementary file 3 — Supplementary Software 1 [file 41467_2021_26196_MOESM3_ESM.zip › Humans-monitor-LP-2.0/images/s2_16_thumb.png]

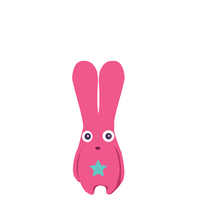

Supplement: Supplementary file 3 — Supplementary Software 1 [file 41467_2021_26196_MOESM3_ESM.zip › Humans-monitor-LP-2.0/images/s2_17_thumb.png]

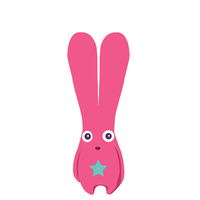

Supplement: Supplementary file 3 — Supplementary Software 1 [file 41467_2021_26196_MOESM3_ESM.zip › Humans-monitor-LP-2.0/images/s2_18_thumb.png]

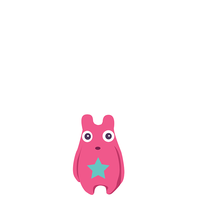

Supplement: Supplementary file 3 — Supplementary Software 1 [file 41467_2021_26196_MOESM3_ESM.zip › Humans-monitor-LP-2.0/images/s2_19_thumb.png]

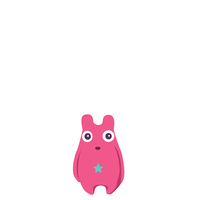

Supplement: Supplementary file 3 — Supplementary Software 1 [file 41467_2021_26196_MOESM3_ESM.zip › Humans-monitor-LP-2.0/images/s2_1_thumb.png]

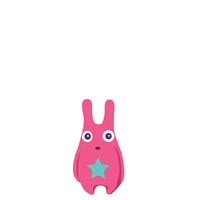

Supplement: Supplementary file 3 — Supplementary Software 1 [file 41467_2021_26196_MOESM3_ESM.zip › Humans-monitor-LP-2.0/images/s2_20_thumb.png]

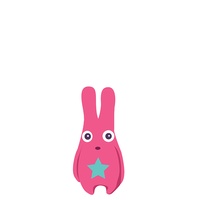

Supplement: Supplementary file 3 — Supplementary Software 1 [file 41467_2021_26196_MOESM3_ESM.zip › Humans-monitor-LP-2.0/images/s2_21_thumb.png]

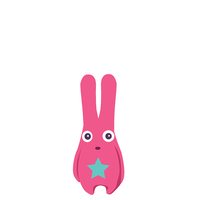

Supplement: Supplementary file 3 — Supplementary Software 1 [file 41467_2021_26196_MOESM3_ESM.zip › Humans-monitor-LP-2.0/images/s2_22_thumb.png]

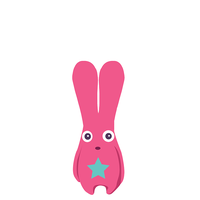

Supplement: Supplementary file 3 — Supplementary Software 1 [file 41467_2021_26196_MOESM3_ESM.zip › Humans-monitor-LP-2.0/images/s2_23_thumb.png]

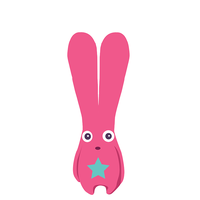

Supplement: Supplementary file 3 — Supplementary Software 1 [file 41467_2021_26196_MOESM3_ESM.zip › Humans-monitor-LP-2.0/images/s2_24_thumb.png]

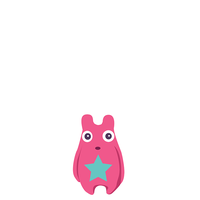

Supplement: Supplementary file 3 — Supplementary Software 1 [file 41467_2021_26196_MOESM3_ESM.zip › Humans-monitor-LP-2.0/images/s2_25_thumb.png]

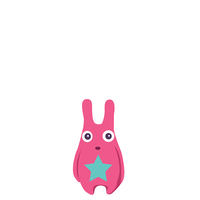

Supplement: Supplementary file 3 — Supplementary Software 1 [file 41467_2021_26196_MOESM3_ESM.zip › Humans-monitor-LP-2.0/images/s2_26_thumb.png]

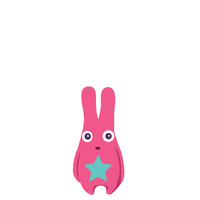

Supplement: Supplementary file 3 — Supplementary Software 1 [file 41467_2021_26196_MOESM3_ESM.zip › Humans-monitor-LP-2.0/images/s2_27_thumb.png]

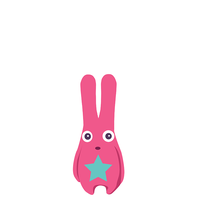

Supplement: Supplementary file 3 — Supplementary Software 1 [file 41467_2021_26196_MOESM3_ESM.zip › Humans-monitor-LP-2.0/images/s2_28_thumb.png]

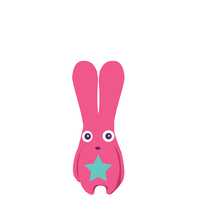

Supplement: Supplementary file 3 — Supplementary Software 1 [file 41467_2021_26196_MOESM3_ESM.zip › Humans-monitor-LP-2.0/images/s2_29_thumb.png]

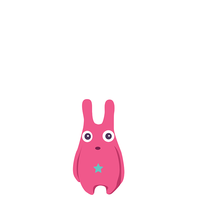

Supplement: Supplementary file 3 — Supplementary Software 1 [file 41467_2021_26196_MOESM3_ESM.zip › Humans-monitor-LP-2.0/images/s2_2_thumb.png]

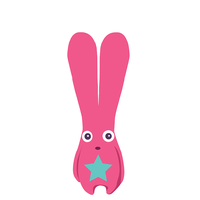

Supplement: Supplementary file 3 — Supplementary Software 1 [file 41467_2021_26196_MOESM3_ESM.zip › Humans-monitor-LP-2.0/images/s2_30_thumb.png]

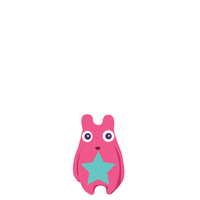

Supplement: Supplementary file 3 — Supplementary Software 1 [file 41467_2021_26196_MOESM3_ESM.zip › Humans-monitor-LP-2.0/images/s2_31_thumb.png]

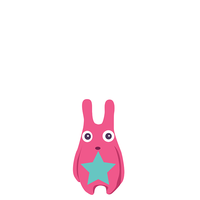

Supplement: Supplementary file 3 — Supplementary Software 1 [file 41467_2021_26196_MOESM3_ESM.zip › Humans-monitor-LP-2.0/images/s2_32_thumb.png]

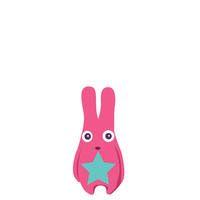

Supplement: Supplementary file 3 — Supplementary Software 1 [file 41467_2021_26196_MOESM3_ESM.zip › Humans-monitor-LP-2.0/images/s2_33_thumb.png]

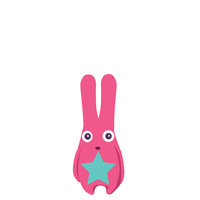

Supplement: Supplementary file 3 — Supplementary Software 1 [file 41467_2021_26196_MOESM3_ESM.zip › Humans-monitor-LP-2.0/images/s2_34_thumb.png]

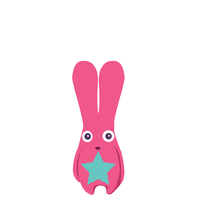

Supplement: Supplementary file 3 — Supplementary Software 1 [file 41467_2021_26196_MOESM3_ESM.zip › Humans-monitor-LP-2.0/images/s2_35_thumb.png]

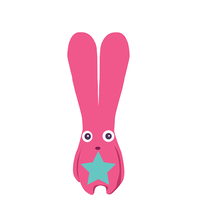

Supplement: Supplementary file 3 — Supplementary Software 1 [file 41467_2021_26196_MOESM3_ESM.zip › Humans-monitor-LP-2.0/images/s2_36_thumb.png]

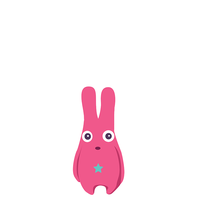

Supplement: Supplementary file 3 — Supplementary Software 1 [file 41467_2021_26196_MOESM3_ESM.zip › Humans-monitor-LP-2.0/images/s2_3_thumb.png]

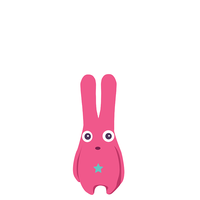

Supplement: Supplementary file 3 — Supplementary Software 1 [file 41467_2021_26196_MOESM3_ESM.zip › Humans-monitor-LP-2.0/images/s2_4_thumb.png]

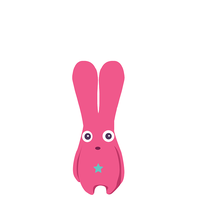

Supplement: Supplementary file 3 — Supplementary Software 1 [file 41467_2021_26196_MOESM3_ESM.zip › Humans-monitor-LP-2.0/images/s2_5_thumb.png]

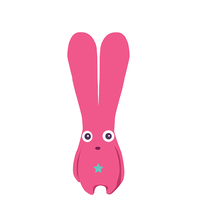

Supplement: Supplementary file 3 — Supplementary Software 1 [file 41467_2021_26196_MOESM3_ESM.zip › Humans-monitor-LP-2.0/images/s2_6_thumb.png]

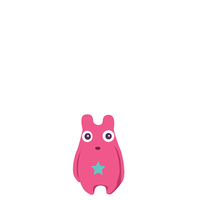

Supplement: Supplementary file 3 — Supplementary Software 1 [file 41467_2021_26196_MOESM3_ESM.zip › Humans-monitor-LP-2.0/images/s2_7_thumb.png]

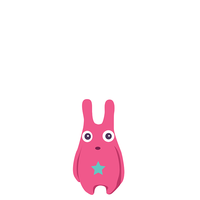

Supplement: Supplementary file 3 — Supplementary Software 1 [file 41467_2021_26196_MOESM3_ESM.zip › Humans-monitor-LP-2.0/images/s2_8_thumb.png]

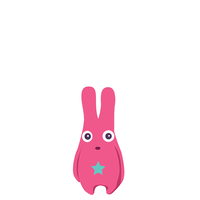

Supplement: Supplementary file 3 — Supplementary Software 1 [file 41467_2021_26196_MOESM3_ESM.zip › Humans-monitor-LP-2.0/images/s2_9_thumb.png]

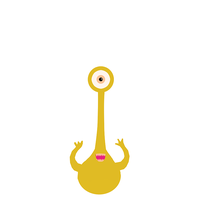

Supplement: Supplementary file 3 — Supplementary Software 1 [file 41467_2021_26196_MOESM3_ESM.zip › Humans-monitor-LP-2.0/images/s3_10_thumb.png]

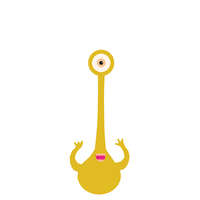

Supplement: Supplementary file 3 — Supplementary Software 1 [file 41467_2021_26196_MOESM3_ESM.zip › Humans-monitor-LP-2.0/images/s3_11_thumb.png]
