# Supplementary material for: Humans monitor learning progress in curiosity-driven exploration
Source: Nat Commun. 2021 Oct 13;12:5972. doi: 10.1038/s41467-021-26196-w (PMC8514490; doi:10.1038/s41467-021-26196-w)
Supplement: Supplementary file 4 — Reporting Summary [file 41467_2021_26196_MOESM4_ESM.pdf]

nature research

Corresponding author(s): Alexandr Ten

Last updated by author(s): Jul 5, 2021

Reporting Summary

Nature Research wishes to improve the reproducibility of the work that we publish. This form provides structure for consistency and transparency in reporting. For further information on Nature Research policies, see our Editorial Policies and the Editorial Policy Checklist.

Statistics

For all statistical analyses, confirm that the following items are present in the figure legend, table legend, main text, or Methods section.

n/a	Confirmed	
☐	☒	The exact sample size (*n*) for each experimental group/condition, given as a discrete number and unit of measurement
☐	☒	A statement on whether measurements were taken from distinct samples or whether the same sample was measured repeatedly
☐	☒	The statistical test(s) used AND whether they are one- or two-sided<br>*Only common tests should be described solely by name; describe more complex techniques in the Methods section.*
☐	☒	A description of all covariates tested
☐	☒	A description of any assumptions or corrections, such as tests of normality and adjustment for multiple comparisons
☐	☒	A full description of the statistical parameters including central tendency (e.g. means) or other basic estimates (e.g. regression coefficient) AND variation (e.g. standard deviation) or associated estimates of uncertainty (e.g. confidence intervals)
☐	☒	For null hypothesis testing, the test statistic (e.g. *F*, *t*, *r*) with confidence intervals, effect sizes, degrees of freedom and *P* value noted<br>*Give P values as exact values whenever suitable.*
☒	☐	For Bayesian analysis, information on the choice of priors and Markov chain Monte Carlo settings
☒	☐	For hierarchical and complex designs, identification of the appropriate level for tests and full reporting of outcomes
☒	☐	Estimates of effect sizes (e.g. Cohen's *d*, Pearson's *r*), indicating how they were calculated

Our web collection on statistics for biologists contains articles on many of the points above.

Software and code

Policy information about availability of computer code

Data collection	javascript and psiturk.js api
Data analysis	Python 3.6 (including libraries : jupyter 1.0.0, matplotlib 3.2.2, numpy 1.19.0, pandas 0.24.1, scipy 1.5.1, statsmodels 0.11.1, seaborn 0.11.0,), R 3.5.0 (including libraries : contrast 0.22, emmeans 1.4, MASS 7.3.51.5, tidyverse 1.3.0, rstatix 0.6.0)<br><br>Data analysis and visualization source code is available at https://doi.org/10.5281/zenodo.5068243

For manuscripts utilizing custom algorithms or software that are central to the research but not yet described in published literature, software must be made available to editors and reviewers. We strongly encourage code deposition in a community repository (e.g. GitHub). See the Nature Research guidelines for submitting code & software for further information.

Data

Policy information about availability of data

All manuscripts must include a data availability statement. This statement should provide the following information, where applicable:

- Accession codes, unique identifiers, or web links for publicly available datasets
- A list of figures that have associated raw data
- A description of any restrictions on data availability

The datasets generated during and/or analyzed during the current study are available in the Open Science Framework repository, https://osf.io/k2yur/

Field-specific reporting

Please select the one below that is the best fit for your research. If you are not sure, read the appropriate sections before making your selection.

☐ Life sciences ☒ Behavioural & social sciences ☐ Ecological, evolutionary & environmental sciences

For a reference copy of the document with all sections, see nature.com/documents/nr-reporting-summary-flat.pdf

Behavioural & social sciences study design

All studies must disclose on these points even when the disclosure is negative.

Study description	People completed an online task lasting for 250 trials (typically under 1 hour) in which they had to repeatedly select between several categorization tasks and provide a binary classification response for the selected task.
Research sample	Our sample prior to exclusions consisted of 400 individuals (female = 208, male = 187, other = 5), aged between 19 and 71 years (median = 34 years). All participants were Amazon MTurk workers. The sample is representative of Amazon MTruk workers. The sample was selected due to its convenience, specifically, the easy access to a large population of adult participants.
Sampling strategy	The sampled participants (N = 400) were recruited from the pool of Amazon MTurk workers. Each participant was randomly assigned to their experimental condition. Due to the novelty of our task, the specific research questions considered, and in particular, our focus on computational modeling, we did not rely on an a priori power analysis to determine the optimal sample size. Rather, we collected reasonably large samples for each experimental group (N = 199 and N = 201) to account for potential individual differences and for possible data exclusions. The sample sizes were determined prior to data collection and all data was collected prior to the reported analyses.
Data collection	Participants completed the behavioral task remotely over the Internet. Participants were instructed to complete the task on their own in a quiet environment and eliminate external distractions (e.g. turn off cell phones, TV sets, music players, etc). Participant responses and related variables were collected using the custom code written in javascript and psiturk.js API. Group assignment was performed by computer randomization, so the researchers had no control over the condition assignment of individual participants.
Timing	May 22 and May 23, 2017
Data exclusions	Eighteen (18) participants were excluded from the analyses, based on a statistical screening procedure aimed to identify non-compliance with the experimental instructions (details provided in the Methods section of the article)
Non-participation	Although it was possible to drop out or decline participation at will, none of the participants dropped out or declined participation.
Randomization	Participants were randomly assigned to their experimental groups

Reporting for specific materials, systems and methods

We require information from authors about some types of materials, experimental systems and methods used in many studies. Here, indicate whether each material, system or method listed is relevant to your study. If you are not sure if a list item applies to your research, read the appropriate section before selecting a response.

Materials & experimental systems

n/a	Involved in the study	
☒	☐	Antibodies
☒	☐	Eukaryotic cell lines
☒	☐	Palaeontology and archaeology
☒	☐	Animals and other organisms
☐	☒	Human research participants
☒	☐	Clinical data
☒	☐	Dual use research of concern

Methods

n/a	Involved in the study	
☒	☐	ChIP-seq
☒	☐	Flow cytometry
☒	☐	MRI-based neuroimaging

Human research participants

Policy information about studies involving human research participants

Population characteristics	See above
Recruitment	Participants were recruited online from the pool of Amazon MTurk workers. We are not aware of any biases due to our sampling strategy that could have impacted the results.

Ethics oversight | International Review Board of the University of Rochester

Note that full information on the approval of the study protocol must also be provided in the manuscript.
